# The rice blast resistance gene *Ptr* encodes an atypical protein required for broad-spectrum disease resistance

**DOI:** 10.1038/s41467-018-04369-4

**Published:** 2018-05-23

**Authors:** Haijun Zhao, Xueyan Wang, Yulin Jia, Bastian Minkenberg, Matthew Wheatley, Jiangbo Fan, Melissa H. Jia, Adam Famoso, Jeremy D. Edwards, Yeshi Wamishe, Barbara Valent, Guo-Liang Wang, Yinong Yang

**Affiliations:** 1 USDA ARS Dale Bumpers National Rice Research Center, Stuttgart, AR 72160 USA; 2 University of Arkansas Rice Research and Extension Center, Stuttgart, AR 72160 USA; 3 Department of Plant Pathology and Environmental Microbiology, Huck Institute of the Life Sciences, The Pennsylvania State University, University Park, PA, 16802 USA; 4 Department of Plant Pathology, The Ohio State University, Columbus, OH 43210 USA; 5 Louisiana State University Agriculture Center, 1373 Caffey Road, Rayne, LA 70578 USA; 6 Kansas State University, 1712 Claflin Rd, 4024 Throckmorton Ctr., Manhattan, KS 66506 USA; 7 Present Address: Innovative Genomics Institute, University of California, Berkeley, CA 94720 USA

## Abstract

Plant resistance genes typically encode proteins with nucleotide binding site-leucine rich repeat (NLR) domains. Here we show that *Ptr* is an atypical resistance gene encoding a protein with four Armadillo repeats. *Ptr* is required for broad-spectrum blast resistance mediated by the NLR *R* gene *Pi-ta* and by the associated *R* gene *Pi-ta2*. *Ptr* is expressed constitutively and encodes two isoforms that are mainly localized in the cytoplasm. A two base pair deletion within the *Ptr* coding region in the fast neutron-generated mutant line M2354 creates a truncated protein, resulting in susceptibility to *M. oryzae*. Targeted mutation of *Ptr* in a resistant cultivar using CRISPR/Cas9 leads to blast susceptibility, further confirming its resistance function. The cloning of *Ptr* may aid in the development of broad spectrum blast resistant rice.

## Abstract (web summary)

Genes that confer resistance to plant pathogens such as rice blast disease typically encode NLR-type receptor proteins. Here, Zhao et al. describe a new resistance locus that encodes a non-NLR Armadillo repeat protein required for broad-spectrum resistance in rice.

## Introduction

Plants have evolved a multifaceted, sophisticated defense response to microbial pathogens carrying effectors, as well as pathogen-associated molecular patterns (PAMP). The first tier of plant defense is PAMP-triggered immunity (PTI) mediated by pattern recognition receptors and occurs during pathogen attachment and the early phase of host-pathogen interactions. The second tier of plant defense is the effector-triggered immunity (ETI) mediated by plant resistance (*R*) genes, most of which encode cytoplasmic proteins with nucleotide binding site-leucine-rich repeat (NLR) domains^1^. In contrast to the structural conservation of most R proteins, effectors are highly diverse molecules with few conserved signatures. Despite the molecular diversity of pathogen effectors, plants have evolved various sophisticated mechanisms to detect intrusion of effectors and initiate robust disease resistance responses. A better understanding of these defense mechanisms will accelerate the translation of this knowledge into practical use in plant breeding and agriculture.

Rice is one of the most important foods for humanity and is widely consumed in the world. Blast disease of rice plants caused by the filamentous fungus *Magnaporthe oryzae* (synonymous with *Pyricularia oryzae*) is one of the most damaging diseases and frequently causes severe reduction of rice yield. The global annual crop loss due to blast was estimated at $66 billion and is enough to feed 60 million people^2^. Even without undergoing sexual reproduction, *M. oryzae* is capable of rapid genetic changes through active transposable elements, which can lead to a loss of avirulence (*AVR*) genes, resulting in the evasion of host defense and occurrence of rice blast disease^3^. So far over 100 major blast *R* genes have been identified and 30 of them have been molecularly cloned^4^. Major blast *R* genes, *Pi-ta*/*Pi-ta2*, *Pi-z*, *Pi-b*, and *Pi-k*/*h*/*m*/*s*, have also been deployed in some US rice varieties^5^. Nearly all of the cloned blast *R* genes encode NLR proteins that may directly or indirectly interact with fungal effectors to trigger ETI^1^. Noticeably, a cluster of blast *R* genes on rice chromosome 12 has been used to effectively reduce blast disease in *indica* germplasm worldwide since 1960. In the United States, the *tropical japonica* cultivar Katy has been widely used in breeding programs as a source of the *Pi-ta* resistance complex which includes three *R* genes, *Pi-ta*, *Pi-ta2*, and *Ptr*, in a region of suppressed recombination^6–8^. This complex was introgressed from the variety Tetep and has been shown to be effective in preventing infections by a wide range of *M. oryzae* strains in the US over two decades^7,8^. Compared to *Pi-ta*^5^, the *Pi-ta2* gene confers higher levels of resistance, and it confers broader-spectrum resistance to all the same strains as *Pi-ta* plus additional strains^9^. Thus far, all rice varieties reported to contain *Pi-ta2* also contain *Pi-ta*, and they are resistant to fungal strains with either *AVR-Pita* or another uncharacterized *Pi-ta2*-specific *AVR* gene^9–11^. The *Ptr* gene was identified in a fast neutron induced mutant (M2354) of Katy that still carries *Pi-ta*, but is susceptible to *M. oryzae* strains with *AVR-Pita*^12^. Subsequent analysis of a genetic cross between Katy and M2354 revealed a linked locus, *Ptr*, located in a linkage block near the centromere of Chromosome 12 and is presumably required for both *Pi-ta* and *Pi-ta2* mediated disease resistance^12^.

Here we show that *Ptr* encodes two isoforms, each with four Armadillo (ARM) repeats. We find that a two base pair (bp) deletion within the *Ptr* protein coding region in the mutant line M2354 produces a truncated protein rendering susceptibility to *M. oryzae*. The resistance function of *Ptr* is further confirmed by showing that targeted mutation of *Ptr* in a resistant cultivar using CRISPR/Cas9 leads to blast susceptibility. More importantly, our genetic analysis suggests that Ptr, a non-NLR protein, functions in broad-spectrum blast resistance independent of *Pi-ta*, providing a strategy for developing blast resistance rice cultivars.

## Results

### Molecular cloning of the *Ptr* gene

**Fig. 1:**
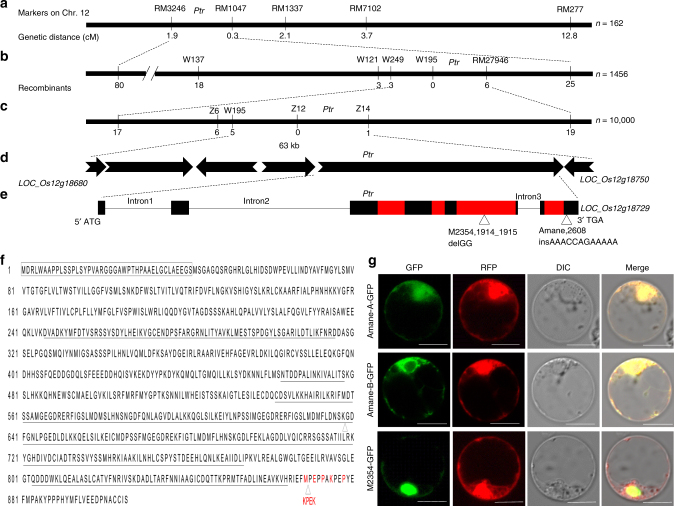
Cloning and characterization of *Ptr*. **a** *Ptr* was mapped between SSR RM3246 and RM1047 on chromosome 12 (Chr. 12). **b**, **c** Fine mapping of *Ptr* to 63 kb. **d** Predicted open reading frames (ORFs) with indicated direction of transcription. **e** Diagram of *Ptr*, mutations identified in M2354 and Amane. Exons are indicated by black filled rectangles. Armadillo (ARM) repeats (Superfamily 1.75) are indicated by the red filled rectangles. Insertion/deletion (InDel) is indicated by triangles. **f** The protein sequence of *Ptr* in Katy. Black rectangle indicates 41 missing amino acids in A isoform (864 amino acids) compared to B isoform (905 amino acids). The ARM repeats are underlined and polymorphic regions are indicated by triangles. Compared to Katy, the 2 bp (GG) deletion of *Ptr* in M2354 resulted in a truncated protein with 645 amino acids, while five different amino acids, as well as a four amino acid (KPEK) insertion in Amane, are indicated in red. **g** Subcellular localization of the Ptr-green fluorescent protein (GFP) fusion proteins in rice protoplasts expressing cytoplasmic/nuclear-localized red fluorescent protein (RFP). Pictures were taken 16-h after protoplast transfection. GFP, RFP, differential interference contrast (DIC), and merged channels are indicated on the top. Scale bars represent 10 μm

To clone the *Ptr* gene, a blast susceptible *indica* rice variety, Amane, containing *Pi-ta* was crossed with Katy (*Pi-ta*/*Pi-ta2*/*Ptr*). The *Ptr* gene was initially mapped to the short arm of chromosome 12 between microsatellite (SSR) markers RM3246 and RM1047 using 162 F_2_ individuals, and its resistance phenotype was confirmed in the F_3_ progenies by inoculating with blast race (isolate) IB-49 (ML1) (Fig. 1a). An additional 1456 F_2_ individuals were genotyped using RM3246, RM27941, and RM1047 to identify 105 recombinants. Four additional SSR markers, W137, W121, W195, and W249, were developed to screen the recombinants along with RM27946 delimiting the region between W249 and RM27946 (Fig. 1b). To fine map *Ptr*, an additional 10,000 F_2_ progeny of the Amane x Katy cross were evaluated to identify five recombinants with SSR marker W195 at one border and one recombinant with cleaved amplified polymorphic sequence (CAPS) marker Z14 at the other border (Fig. 1c, d). The insertion/deletion (InDel) marker Z12 at LOC_Os12g18729 was found to co-segregate with *Ptr*. A comparison of the corresponding genomic region of *Ptr* in Amane and Katy revealed 23 single nucleotide polymorphisms (SNPs) and four InDels that distinguish Katy from Amane. Among them, five SNPs resulting in nonsynonymous mutations and one 12 bp deletion resulting in a four- amino acid deletion defines the functional region of *Ptr* in the fourth exon (Supplementary Table 1).

Rice mutant M2354 carrying *Pi-ta* is susceptible to *M. oryzae* isolates with *AVR-Pita*^12^. DNA sequence alignments between M2354 and Katy of six candidate genes within the 63 kb mapping region revealed only a 2 bp (GG) deletion in the fourth exon of the LOC_Os12g18729 gene, creating a premature stop codon (Fig. 1e). To verify if susceptible *ptr* in M2354 is allelic to that of Amane, 800 F_2_ progeny from crossing Amane with M2354 were phenotyped and all were shown to be blast susceptible, suggesting that *ptr* in M2354 is allelic to *ptr* in Amane. To validate this finding, we analyzed 160 F_3_ progeny of the same cross with InDel markers developed from the 2 bp deletion in M2354 and 12 bp insertion in Amane. All F_3_ lines either heterozygous or homozygous at *ptr* were susceptible, verifying that *ptr* in Amane is allelic to *ptr* in M2354 (Supplementary Table 2).

To determine if the 2 bp deletion in M2354 resulted in a truncated protein that co-segregated with blast susceptibility, the InDel marker Z11, specific to the deletion in M2354, was used to analyze 300 F_2:3_ individuals of the cross between M2354 and Katy. The resulting data showed that all 81 susceptible individuals contain the 2 bp deletion, whereas all resistant individuals lack the deletion (Supplementary Table 2). Taken together these data strongly suggest that locus LOC_Os12g18729 is the *Ptr* gene.

### Function validation of *Ptr* using CRISPR/Cas9

**Fig. 2:**
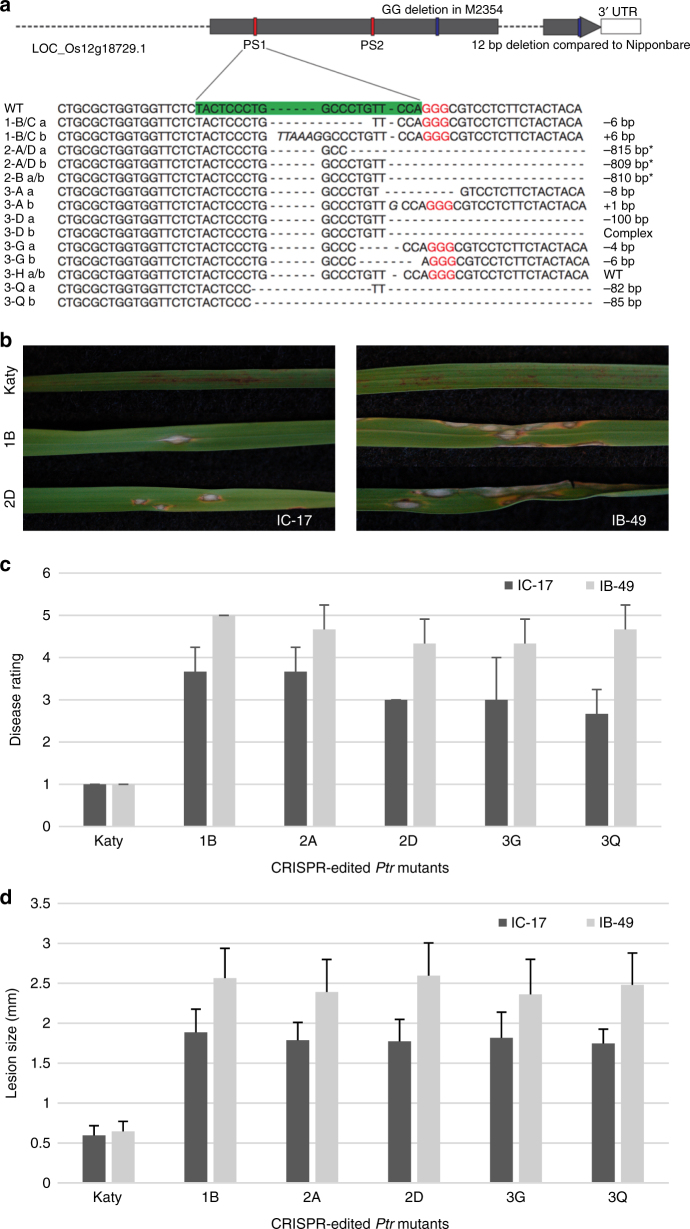
CRISPR/Cas9-mediated mutation of *Ptr* in a resistant cultivar. **a** The *Ptr* gene was targeted with two specific gRNAs to achieve knockouts. CRISPR/Cas9 editing resulted in InDels of differing length or a fragment deletion between the two protospacer (PS) sites are indicated by asterisk (*). **b** Disease symptoms on wild-type Katy and CRISPR-edited *Ptr* mutant leaves after spray-inoculation with *AVR-Pita*-containing strains IB-49 (ML1) or IC-17 (ZN57). **c** Disease rating of Katy and *Ptr* mutant plants at 6 days post-inoculation with 0–5 rating scale where 0–2 is resistant and 3 to 5 is susceptible. **d** Average lesion length on Katy and *Ptr* mutant leaves at 6 days post-inoculation. Error bars indicate standard deviation (s.d.) of average lesion area and disease rating. Data represent means ± s.d. (*n* = 12)

To confirm the function of *Ptr*, we examined whether disruption of *Ptr* in the resistant cultivar Katy would lead to the same susceptible phenotype as the M2354 mutant. Targeted mutation was performed at the LOC_Os12g18729 locus using CRISPR/Cas9. Two sequence-specific guide RNAs (gRNAs) were designed to disrupt the *Ptr* gene sequence in the third exon in front of the 2 bp mutation found in M2354 (Fig. 2a) ensuring that the targeted mutation will affect this region. The targeting sites encompassed an *Eco*NI (protospacer 1) and a *Sac*I site (protospacer 2) to allow rapid evaluation of the mutation sites by PCR-restriction enzyme (PCR-RE) assay (Fig. 2a and Supplementary Fig. 1a). InDels generated by Cas9 typically lead to frame-shift mutations downstream of the targeted site and eventually result in the formation of a premature stop codon. A total of 34 plants from four independent events were obtained and confirmed to carry mostly InDels or a deletion of 810 bp between the two targeting sites (Fig. 2a and Supplementary Fig. 1b). Subsequently, the progeny derived from nine CRISPR-edited mutants (including plant 3H that carried two wild-type alleles and was not a true mutant) were inoculated with two avirulent races (isolate), IB-49 (ML1) or IC-17 (ZN57), of *M. oryzae* carrying *AVR-Pita*^12^. In comparison with Katy, CRISPR-edited *ptr* mutants exhibited a much larger lesion area and had a higher disease rating with both spray- and spot-inoculations (Fig. 2b–d and Supplementary Figs 2 and 3). Therefore, the targeted mutation of *Ptr* in the resistant Katy cultivar led to disease susceptibility to avirulent isolates, further confirming that *Ptr* is required for rice blast resistance.

### *Ptr* function is independent of *Pi-ta*

**Fig. 3:**
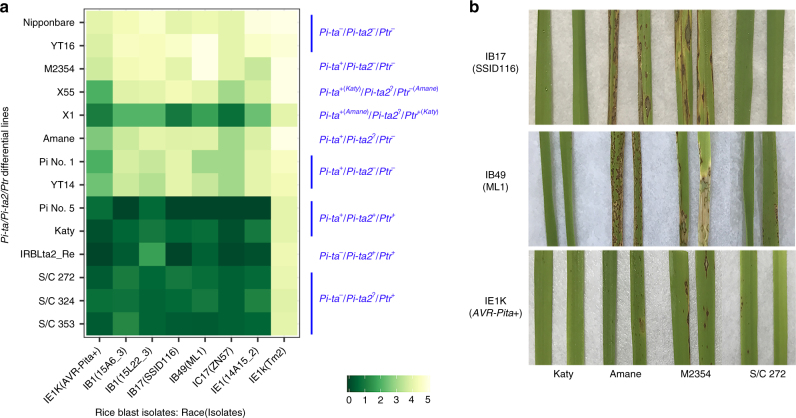
Disease reactions of *Pi-ta*/*Pi-ta2*/*Ptr* differential lines. **a** Disease reactions of *Pi-ta*/*Pi-ta2*/*Ptr* lines to four diverse races/isolates. Disease reaction was evaluated 7 days post-inoculation with 0–5 rating scale where 0–2 is resistant and 3 to 5 is susceptible. Rice varieties with the *Pi-ta*/*Pi-ta2*/*Ptr* genes were susceptible to IE-1K (TM2 lacking *AVR-Pita*) and resistant or moderately resistant to the transformant IE-1K + *AVR-Pita* from O-137^15^. Note: *M. oryzae* races (isolates), IB-1 (15A6_3, 15L22_3), IB-17 (SSID116), IB-49 (ML1), and IE-1 (14A15–2), differentiated the *Pi-ta*-containing rice lines from the rice lines with *Pi-ta2*/*Ptr*. **b** Photographs showing disease reaction of indicated rice lines and races: Katy (*Pi-ta*^*+*^/*Pi-ta2*^*+*/^*Ptr*^*+*^), Amane (*Pi-ta*^+^/Pi-ta2?/Ptr^-^) and M2354 (*Pi-ta*^+^/*Pi-ta2*^-^/*Ptr*^*−*^), and S/C272 (*Pi-ta*^−^/*Pi-ta2?*/*Ptr*^*+*^). Pictures were taken at seven days post-inoculation

The complete susceptibility of M2354 with the truncated ptr protein suggests *Pi-ta* requires *Ptr* to function (Supplementary Table 3). To examine if *Pi-ta* is required for *Ptr* function, we identified three *Ptr*-containing recombinant inbred lines (RILs), S/C272, S/C324, and S/C353, lacking *Pi-ta* and *Pi-b* from the cross of Saber with Cybonnet (Supplementary Table 4). Saber carries *Pi-b* and *Pi-km* but lacks *Pi-ta*/*Pi-ta2*/*Ptr* resistance alleles resulting in susceptibility to isolates carrying *AVR-Pita*. Cybonnet was bred from Katy carrying *Pi-ta*/*Pi-ta2*/*Ptr*. Inoculation of these RILs with fungal isolates lacking *AVR-Pi-km* excluded recognition by *Pi-km*^13^. As expected, S/C272, S/C324, and S/C353 were resistant to the fungal race/isolate IB-49 (ML1) with *AVR-Pita* (Fig. 3a). To verify that resistance in these RILs was associated with the *Ptr* gene, we crossed S/C272 with the blast susceptible rice variety M202 (lacking *Pi-ta*/*Pi-ta2*/*Ptr*) and tested progeny with the *Ptr* gene-specific marker Z12. As shown in Supplementary Table 5, resistance to IB-49 (ML1) co-segregated with the *Ptr* gene in F_2_ progeny suggesting that the *Ptr* gene-mediated resistance can be inherited. Taken together these data suggest that *Ptr* is a *Pi-ta* independent blast *R* gene.

### *Ptr* is required for broad-spectrum resistance

To determine the resistance spectrum of *Ptr* relative to *Pi-ta* and *Pi-ta2*, 389 genetically diverse isolates^14^ collected from the United States were used to inoculate Katy and M2354. The extremely susceptible *japonica* rice cultivar Lijiangxintuanheigu (LTH) with an unknown *R* gene was resistant to only six of these isolates. In contrast, Katy carrying *Pi-ta*, *Pi-ta2*, *Ptr*, and *Pi-ks* was resistant to 348 isolates. Katy mutant M2354 with a truncated *ptr* gene, but still containing *Pi-ta*/*Pi-ta2* and *Pi-ks*, was only resistant to 17 isolates. Therefore, *Ptr* plays a major role in the broad-spectrum resistance associated with the *Pi-ta* resistance complex^12^ (Supplementary Data 1).

To determine the resistance spectrum of rice lines with *Ptr* but without *Pi-ta*, we inoculated S/C272, S/C324, and S/C353 RILs with six *Pi-km* virulent blast races/isolates of IB-1 (15A6_3, 15L22_3), IB-17 (SSID116), IB-49 (ML1), IC-17 (ZN57), and IE-1 (14A15–2). As shown in Fig. 3a, resistance was observed for all six isolates. As a control, resistance was also observed when these RILs were inoculated with AVR-*Pita*-containing strains IE-1K (+*AVR-Pita*), a virulent strain that was transformed with the original reference *AVR-Pita* from blast strain O-137^11,15^. Rice varieties Pi No. 1 and YT14 that only contain *Pi-ta* showed moderate levels of resistance to two strains, IE-1K (*+AVR-Pita*) and IC-17 (ZN57), and rice varieties Nipponbare and YT16 that lack all three genes were susceptible to all strains (Fig. 3a). These data indicate that *Ptr* is required for a broader-spectrum of blast resistance than *Pi-ta* alone.

### Relationship between *Ptr* and *Pi-ta2*

To determine the relationship between *Ptr* and *Pi-ta2*, differential rice varieties with *Pi-ta2* and/or *Pi-ta* with or without various *Ptr* alleles were used for blast inoculations using differential blast races including a virulent race IE-1K (+*AVR-Pita*)^11^ (Fig. 3a, b). DNA sequence analysis showed that *Ptr* haplotypes in these rice varieties with *Pi-ta2* are identical to that of Katy and rice varieties carrying only *Pi-ta* have a different *ptr* haplotype. Eight additional rice varieties known to contain *Pi-ta2*, including Pi No. 5 and IRBLta2_Re, share the Katy *Ptr* resistance allele containing the 12 bp deletion (Supplementary Table 6). The *Pi-ta2*/*Ptr* varieties Katy, Pi No. 5 and IRBLta2_Re show stronger, broader-spectrum resistance than varieties that contain only *Pi-ta* (Fig. 3a). Based on this data it is not possible to distinguish between *Ptr* and *Pi-ta2*.

To further define the resistance spectrum of *Ptr*, 10 differential varieties/lines were repeatedly inoculated with 18 diverse contemporary blast races/isolates (Table 1). Rice variety IR64 which contains the *Pi-ta* resistance complex plus additional blast *R* genes, was used as a resistant control^6^. LTH, the recurrent parent for IRBLta2_Re with *Pi-ta2*, and M202 were used as susceptible controls. The resistance frequencies of *Pi-ta* rice varieties YT14 and Pi No.1 were 27.8% and 38.9%, respectively. The resistance frequency of S/C272 with *Ptr* but without *Pi-ta* was 77.8%. The resistance frequency of Katy (88.9%) (*Ptr*/*Pi-ta2* + *Pi-ta*) is identical to that of two diverse *Pi-ta2*-containing rice varieties, IRBLta2_Re and Pi No.5. Therefore, based on assays with diverse fungal strains, the *Ptr* resistance spectrum is highly similar to that of *Pi-ta2*.

**Table 1:** *Ptr* without *Pi-ta* confers similar blast resistance spectra to that of *Pi-ta2*

Race	Isolates^a^	S/C 272 (*Ptr*)^b^	IRBLta2_Re (*Pi-ta2*)^c^	Pi No. 5 (*Pi-ta2*)^c^	Katy *(Ptr*/*Pi-ta2)*^c^	YT14 (*Pi-ta*)^d^	Pi No. 1 (*Pi-ta*)^d^	M2354 (*ptr*)^d,e^	M202 (*ptr*)^e^	LTH (*ptr*)^e^	IR64 (*Pi-ta* + *R*)^f^
IA-1	15A16_1	3	1.4	3.5	2	4	3.3	5	5	4.5	0
IA-113	15A5_2	3	1.8	0	1.7	4	4.8	4.5	4.5	4.3	0
IB-1	14A30_6	2.5	2	2	2.5	4	4.5	5	5	5	0.3
IB-1	14L48_4	3	0.5	0.7	0.3	4.8	4	5	5	5	2
IB-1	15A6_3	1.1	0.3	1.3	0.3	4	4.5	4.3	5	4.3	0
IB-1	15L15_1	0	3.3	1.3	3	4.5	4.5	5	5	5	2
IB-1	15L22_3	0.6	1.7	0.5	1	4	3.5	4.5	4.5	5	1
IB-17	SSID116	1	0	1	1	4	4	4	4	5	0.3
IB-17	14A18_6	2.5	0.3	1.7	1	2.8	1	5	5	5	0
IB-17	14L42_3	0	0	0	0	3.5	2	5	5	4.5	0
IB-17	15A27_1	0.5	0	0	1	1.7	1.7	5	3.8	5	0.5
IB-17	15L3_1	0	1	0	0	1.8	2.8	4.5	5	4.5	0
IB-25	SSID60	0	0	0	0	3	1	5	4	5	1
IB-49	15A25_1	1	0	0	0	1.8	4	5	3	4	0
IC-17	14L71_1	1	0.3	0	0	1.3	1.5	5	4	5	0.5
IE-1	14A15_2	0.7	1.5	0	1	4	4	4.5	4	4	0.5
IE-1	15A23_2	1	0	0	0	3.3	1.8	5	4	4.2	0
IE-1K	TM2	4.1	5	4	4	4.3	4.5	5	4.5	4.3	1.7
Total # of *R*		14	16	16	16	5	7	0	0	0	18
% of *R*		77.8	88.9	88.9	88.9	27.8	38.9	0	0	0	100

Italicization in table: *Pi-ta*, *Pi-ta2*, and *Ptr*/*ptr* are resistance genes; *R* in the column header stands for resistance gene

^a^ Selected diverse contemporary blast races/isolates (14) were used to evaluate blast reaction using a 0–5 scale standard: 0–2, resistant and 3–5, susceptible. *R* indicates resistant reaction based on the average of 5 to 20 plants

^b^ This S/C RIL contains *Ptr* without *Pi-ta*

^c^ These rice varieties contains both *Pi-ta2* and *Pi-ta*

^d^ These rice varieties only contain *Pi-ta*

^e^ These rice varieties do not carry resistant *Ptr* haplotypes

^f^ This rice variety contains *Pi-ta* and other blast *R* genes as a resistant control

We tested if mutation of *Ptr* impacted recognition of both *AVR* genes previously associated with *Pi-ta2*. Specifically, these are *AVR-Pita* and a second uncharacterized *AVR* gene identified by isogenic *AVR* strain/virulent mutant pairs^16^. Together with known *Pi-ta* and *Pi-ta2* rice varieties, mutant M2354 with the truncated *ptr* gene was assayed for resistance to the diagnostic strain pairs for *AVR-Pita* and the second *Pi-ta2 AVR* gene (Supplementary Table 3). Mutation of *Ptr* compromises recognition and responses mediated by both *Pi-ta2* associated *AVR* genes. Again, *Ptr* and *Pi-ta2* are indistinguishable based on the data we have obtained so far.

### Expression analysis of the *Ptr* and *Pi-ta* genes

The *Ptr* gene was expressed constitutively in all plant parts with transient induction within 16 h in both compatible (Amane) and incompatible (Katy) interactions (Supplementary Fig. 4a, b). Similarly, the *Pi-ta* gene was expressed constitutively in Katy and Amane with transient induction at 16 h in both compatible and incompatible interactions. In susceptible M2354, the highest transcript accumulation was at 24 h post-inoculation (hpi) for *Pi-ta* and at 48 hpi for *ptr* suggesting that both genes have roles during disease development (Supplementary Fig. 4b, c).

### Subcellular location of the Ptr protein

The *Pt*r gene is located 211 kb away from *Pi-ta* and the genomic sequence of *Ptr* in Katy is 7713 bp. Comparison of cDNAs predicted two or three introns producing two putative proteins with 864 (A isoform) and 905 (B isoform) amino acids, respectively (Fig. 1f). Significant matches of an Armadillo (ARM) repeat domain without a U-box indicate *Ptr* may encode a non-typical E3 ligase (Fig. 1f). The absence of a nuclear localization signal in the Ptr protein suggests that Ptr may be localized in the cytoplasm of plant cells. To test this prediction, we made in-frame fusions of the coding regions of both Ptr isoforms (2592 bp and 2715 bp, without stop codon) with enhanced green fluorescent protein (EGFP) gene to produce Katy isoforms A and B: C-terminal EGFP fusion proteins for transfection into rice protoplasts. However, fluorescence signals from Katy A and B: C-terminal GFP were weakly visible (Supplementary Fig. 5). Consistently, the same fusion protein induced cell death in *Nicotiana* leaves via *Agrobacterium*-mediated expression, suggesting that this fusion protein is toxic to plant cells. Then, we made an in-frame fusion protein with a susceptible ptr protein from Amane. Subsequently, C-terminal GFP fusion proteins were produced from two ptr isoforms from Amane, namely, Amane A isoform (868 amino acids = Katy A isoform +4 amino acid insertion) and Amane B isoform (909 amino acids =Katy B isoform +4 amino acid insertion), as well as from the truncated ptr protein in M2354 (B isoform-645 amino acids). The resulting three constructs were transfected into rice protoplasts using the whole-cell-localized red fluorescent protein (RFP) as a control. The Amane-A fusion protein without the 41 N- terminal amino acids was localized in the whole cell, whereas the Amane-B fusion protein was mainly localized in the cytoplasm. In contrast, the truncated M2354:GFP fusion protein localized mainly in the nucleus (Fig. 1g), suggesting that proper cytoplasmic-nuclear localization of Ptr may be important for its resistance function.

### Biochemical analysis of the Ptr protein

*Ptr* encodes the ARM repeat domain found in E3 ligases known to be involved in plant *R* gene-mediated resistance^17^. An ubiquitination assay was performed with Maltose Binding Protein (MBP) in-frame fused with the B isoform of  the Ptr protein from Katy, Amane, and M2354, respectively. The fusion protein could not be expressed in bacteria. Fusion proteins of Ptr without a predicted transmembrane domain, 234 amino acids at N terminus, were made. As shown in Supplementary Fig. 6a, fusion proteins with expected sizes were purified for assays. Polyubiquitination occurred with positive control AvrPtoB, an E3 ligase protein as expected^18^; however, this event was not observed for Ptr from Katy despite E2 adducts being seen with and without Ptr (Supplementary Fig. 6b). To eliminate E2-ubiquitin adducts, we performed the same assays with a total of 10× dilutions of four different E2, one of which was shown in Supplementary Fig. 6c. The absences of polyubiquitination signals at the sites for the predicted Ptr proteins from Katy, M2354, and Amane (Supplementary Fig. 6d) suggest that Ptr is not an E3 ligase.

### Natural variation at the *Ptr* locus

In the absence of E3 ligase activity, we reasoned that the structural integrity of the Ptr protein including the ARM repeat may be important for initiating defense responses. However, we identified five nonsynonymous substitutions and a 12 bp deletion in the fourth exon separating resistant *Ptr* in Katy and susceptible *ptr* in Amane outside of the ARM repeat domain. We then focused on this region of the *Ptr* gene (Supplementary Table 1). To validate the 12 bp deletion, we sequenced the fourth exon of *Ptr* in 13 resistant and 15 susceptible rice varieties (Supplementary Table 6). Noticeably, the 12 bp InDel and its two nearby nonsynonymous SNPs, resulting in a four amino acid deletion (KPEK) and amino acid alterations at M869K and E871K in resistant varieties, distinguish blast resistant from susceptible varieties (Supplementary Table 6). In contrast, the remaining SNP sites, existing in both resistant and susceptible varieties, do not distinguish their disease reactions. To rule out the presence of polymorphic sequences in other exons we sequenced coding regions of a few selected *Ptr* haplotypes (Table 2). Besides the deletion of four amino acids, nucleotides at positions 6925 and 6930 altered amino acids from M to K (at 869 aa sequence) and E to K (at 871 aa sequence), respectively, correlated with disease resistance specificity.

**Table 2:** Sequence analyses of the *Ptr* gene in seven differential rice varieties/lines, and their disease reactions

Variety/line	*Pi-ta*/*Pi-ta2*	Nonsynonymous substitutions within CDS of the *Ptr* gene												Phenotype to IB-49 (ML1)
		+6925^a^	+6927	+6928	+6930	+6936	+6943	+6952	+6963	+6966	+6978	+6991	+7003	
Katy	+/+^b^	T/M^c^	–	C/P	G/E	C/P	A/K	C/P	A/M	C/P	C/P	A/Y	T/V	R^d^
Pi No. 4	+/+	T/M	–	C/P	G/E	C/P	A/K	C/P	A/M	C/P	C/P	A/Y	T/V	R
Pi No. 1	+/−	A/K	AAACCAGAAAAA/KPEK	C/P	A/K	C/P	A/K	C/P	A/M	C/P	C/P	A/Y	T/V	S
Amane	+/?	A/K	AAACCAGAAAAA/KPEK	C/P	A/K	G/A	G/R	T/L	A/M	C/P	C/P	A/Y	T/V	S
YT16	−/−	A/K	AAACCAGAAAAA/KPEK	C/P	A/K	G/A	G/R	T/L	G/V	A/T	A/T	T/F	G/G	S
BHA	−/−	A/K	AAACCAGAAGAA/KPEE	A/Q	A/K	G/A	G/R	T/L	G/V	A/T	A/T	T/F	G/G	S
Nipponbare	−/−	A/K	AAACCAGAAAAA/KPEK	C/P	A/K	G/A	G/R	T/L	G/V	A/T	A/T	T/F	G/G	S

Italicization in table: *Pi-ta*, *Pi-ta2*, and *Ptr* are resistance genes

^a^ The SNP positions were noted based on the Katy *Ptr* genomic DNA (Genbank accession number MG385185) starting from the start codon ATG in Katy

^b^ Symbols ‘+’, ‘−’, and ‘?’ indicate if the gene is absent, present, and unknown, respectively

^c^ Nucleotide/amino acid

^d^ R indicates resistance and S indicates susceptible evaluated seven days post-inoculation with indicated race (isolate), respectively. The details of sequence assemblies of the coding region can be found at Supplementary Fig. 7

**Fig. 4:**
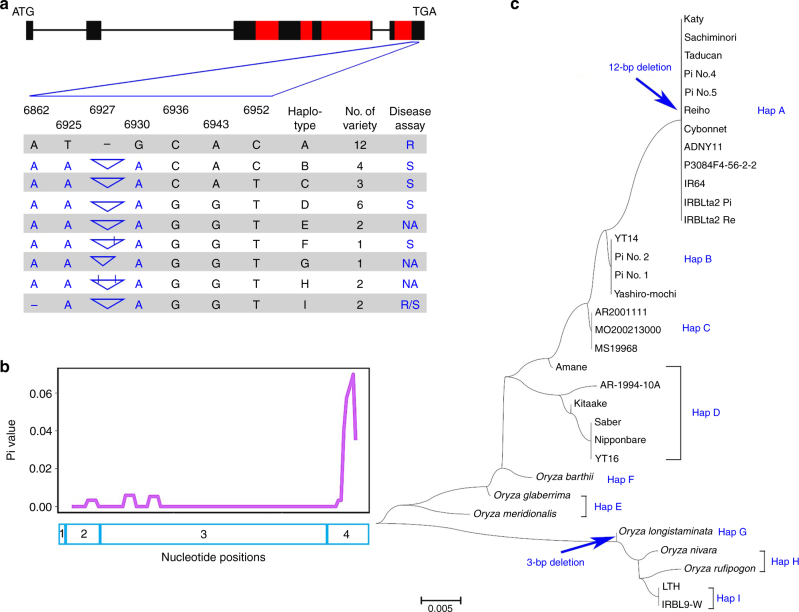
*Ptr* gene variation within *Oryza* species. The DNA sequences from the fourth exon of *Ptr* in cultivated rice and wild rice relatives were used to construct a phylogenetic tree. All the DNA sequences of the wild rice relatives were downloaded from the Gramene website (www.gramene.org). **a** Natural variation in *Ptr*. The haplotype was determined based on five nonsynonymous and 12 bp InDel differences between Katy and Amane in addition to one InDel in front of a 12 bp InDel resulting in a frame-shift of *Ptr*. Exons are indicated by black filled rectangles and Armadillo (ARM) repeats (Superfamily 1.75) are indicated by the red filled rectangles. In the table, the triangles indicate the insertion and width of triangles indicate the insertion size. The black lines in the triangles indicate that the nonsynonymous substitution occurred inside of the 12 bp. NA means data is not available. **b** Sliding window of nucleotide variations in the *Ptr* ORF. The blue rectangles indicate the number and position of each exon. **c** Phylogenetic tree of *Ptr* in *Oryza* species (Supplementary Fig. 8). Hap A corresponds to *Pi-ta*/*Pi-ta2*/*Ptr*-containing varieties and Hap B corresponds to *Pi-ta*-containing rice varieties. Microscale units are as indicated

We noticed that most nucleotide variations are in the fourth exon of *Ptr* as indicated by significant *Pi* value (Fig. 4a,b and Table 2). To determine if this 12 bp InDel co-segregates with resistance, 400 F_2:3_ individuals resulting from the cross between Amane and Katy were analyzed with the InDel marker Z12. The presence of the 12 bp region was strictly associated with blast susceptibility, whereas the absence of the 12 bp was associated with blast resistance, suggesting that Z12 can be a useful marker for the *Ptr* gene and that amino acid composition in the fourth exon is likely important for *Ptr*-mediated disease resistance (Supplementary Table 2).

### Distribution of the *Ptr* gene in rice germplasm worldwide

**Fig. 5:**
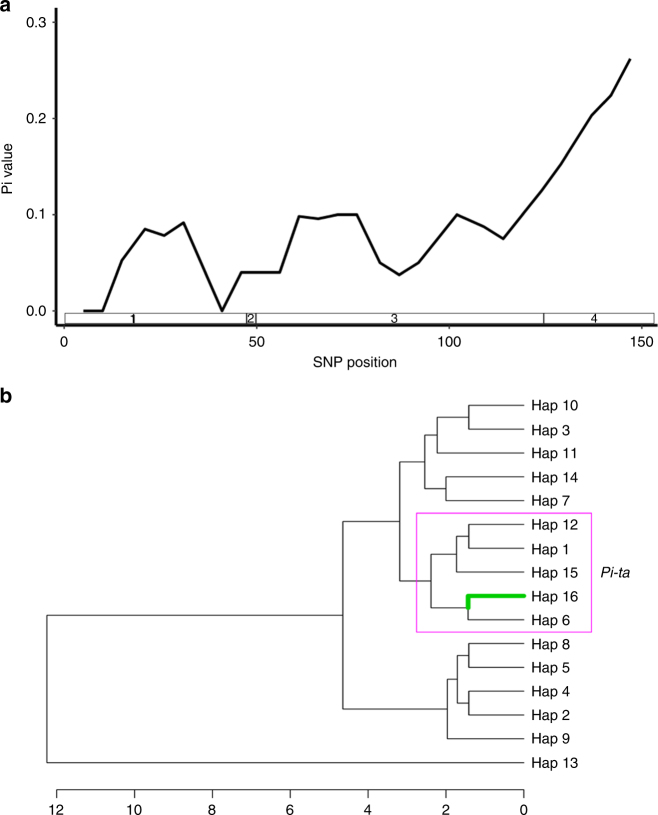
DNA sequence polymorphism of *Ptr* in 3 K rice germplasm. **a** Sliding window analysis of polymorphism with SNPs from indicated coding regions showed in the bars. *x*-axis is the total number for SNPs in exons. **b** Phylogenetic tree of each haplotype (Hap) in supplementary Table 7. Hap 16 has an identical *Ptr* sequence as Katy. Hap 1, Hap 6, Hap 12, Hap 15, and Hap 16 grouped in the pink rectangle contain the identical resistant *Pi-ta* allele in Katy (see Supplementary Data 2 for information on rice varieties for each haplotype and details of SNPs). Evolutionary distance is as indicated on *x*-axis

To deduce the distribution of *Ptr*, we analyzed *Ptr* in IRRI 3 K dataset containing sequencing data of 3000 rice genomes from 89 countries^19,20^ and identified 16 haplotypes including the one containing the deletion of 12 bp in the fourth exon found in 48 mostly *indica* rice varieties (Supplementary Table 7 and Supplementary Data 2). Most importantly, the *ptr* haplotypes containing the 12 bp were found in most rice varieties in the IRRI 3 K dataset, and a few wild rice relatives genotyped by the *Oryza* Map alignment project (OMAP)^21^ suggesting that the resistant *Ptr* haplotype has newly evolved through a 12 bp deletion with its adjacent two nonsynonymous SNP mutations resulting in changes of six amino acids in *indica* subspecies (Fig. 4c and Supplementary Fig. 8). These findings suggest that genomic variation surrounding these InDels determines pathogen signal recognition specificity (Table 2). To validate that the genomic region in the fourth exon determines recognition specificity, we analyzed 16 haplotypes identified in the 3 K dataset (Fig. 5). Sliding window analysis of nucleotide variation based on SNPs for each haplotype revealed that the fourth exon has the most significant DNA sequence variation (Fig. 5a). Phylogenetic analysis showed that all *Pi-ta* containing rice varieties belong to haplotypes 1, 6, 12, 15, and 16 and only haplotype 16 has the resistance allele of *Ptr* (Fig. 5b) suggesting that blast resistance in rice varieties harboring the haplotypes 1, 6, 12, and 15 could be impaired due to the absence of *Ptr* resistance.

### *Ptr* is specifically involved in *Pi-ta*/*Pi-ta2* resistance

Because *Pi-ta* requires the *Ptr* gene to be fully functional, the question was raised if *Ptr* may also be involved in other blast *R* gene-mediated disease resistance. Therefore, we examined if *Ptr* is involved in *Pi9* mediated blast resistance. The monogenic line IRBL9-W carrying *Pi9* was resistant to 14* M. oryzae* races/isolates common to the US including IB-49 (ML1) and IC-17 (ZN57)^22^. We sequenced the *Ptr* allele in IRBL9-W and recurrent parent LTH. A single base deletion occurred before the 12 bp insertion in the fourth exon, resulting in a smaller ptr protein due to a frame-shift and suggesting that *ptr* in IRBL9-W did not compromise the *Pi9* mediated blast resistance (Supplementary Fig. 8 and Supplementary Table 8). Similarly, we showed resistant monogenic lines, IRBLzt-T carrying *Piz-t*, IRBLkm-Ts carrying *Pi-km*, IRBLPi-kh-k3 carrying *Pi-kh*, and IRBLkp-K60 carrying *Pi-kp*, lack the *Ptr* gene as determined with the *Ptr* functional marker (Supplementary Table 8) suggesting that *Ptr* is not involved in *Pizt* and *Pi-km*/*Pi-kh*/*Pi-kp* mediated disease resistance. Previously, we demonstrated that *Ptr* is involved in both *Pi-ta* and *Pi-ta2* (Supplementary Table 3), but not involved in *Pi-ks* mediated blast resistance as evidenced by an incompatible interaction to the AVR race IB54 in M2354^12^. Taken together, we suggest that *Ptr* is specifically involved in *Pi-ta*/*Pi-ta2* mediated blast resistance.

### *Ptr* homologs in other plants

To determine how universal the *Ptr* gene is in plant species, we searched for *Ptr* homologs using protein BLAST program in NCBI database (https://blast.ncbi.nlm.nih.gov/Blast.cgi) and identified *Ptr* orthologs/homologs mainly in monocots including rice, *Sorghum bicolor*, *Brachypodium distachyon*, and *Panicum hallii* (Supplementary Figs 9 and 10). These findings suggest that there exists a unique defense system in monocots.

## Discussion

Rice varieties with the *Pi-ta*, *Pi-ta2*, and *Ptr* genes near the centromere region of chromosome 12 have been effective in reducing blast infection in the US for over two decades, suggesting the existence of an effective signal recognition and transduction mechanism to trigger rice immunity. In the present study, we fine mapped the *Ptr* gene within a 63 kb region nearby *Pi-ta*, and sequence analyses of all six candidate genes in a fast neutron induced susceptible mutant M2354 only identified a 2 bp deletion resulting in a truncated ptr protein. We validated the resistance function of the *Ptr* gene by demonstrating that 34 CRISPR/Cas9 knockout lines carrying different frame-shift mutations in the *Ptr* gene were blast susceptible versus the resistant wild-type parent.

A rice variety with *Pi-ta2* reportedly confers a broad-spectrum blast resistance^10^, that was verified after the monogenic line IRBLta2-Pi carrying *Pi-ta2* was inoculated with 14 commonly found US blast races^22^. In that study, Wang et al.^22^ demonstrated that the monogenic line IRBLta-K1 with *Pi-ta* was moderately resistant to one isolate of IC-17 (7.1%), whereas IRBLta-Pi with *Pi-ta2* was resistant to the most of them (78.6%) suggesting that *Pi-ta2* confers broader-spectrum of blast resistance compared to *Pi-ta*. Previously, we demonstrated that Katy also contains another blast *R* gene, *Pi-ks*, that confers avirulence to the race IB-54 in addition to *Pi-ta* that confers avirulence to isolates with *AVR-Pita*^12^. M2354 carrying *Pi-ks* and a defective *ptr* was extremely susceptible to over 300 diverse rice blast isolates (Supplementary Data 1). M2354 is a fast neutron induced near isogenic line of Katy examined by genome-wide SSR analysis, and no differences of morphological and agronomic traits were observed between Katy and M2354^12^. We suggest that extreme blast susceptibility of mutant-M2354 is due to the loss of function of the *Ptr* gene in the resistant parent Katy. We then identified three S/C RILs with *Ptr* but without *Pi-ta*, and showed that they were resistant to more races/isolates, including that of *Pi-ta*. We demonstrated that *Ptr* is required not only for resistance mediated by *Pi-ta*, but also for a broader spectrum of blast resistance (Fig. 3; Table 2 and Supplementary Tables 3 and 8), though further evidence will be required to test if *Ptr* can confer this resistance independently. Additionally, we showed that a mutation in *Ptr* eliminates recognition of both *AVR-Pita* and the *Pi-ta2*-specific *AVR* gene associated with *Pi-ta2* (Supplemental Table 3). We also showed that all examined rice varieties reportedly carrying *Pi-ta2* contain the identical *Ptr* haplotype (Table 2, Fig. 4 and Supplementary Table 6). *Ptr* and *Pi-ta2* are indistinguishable based on this data though further research will be required to determine if they are the same gene.

We found no evidence that the *Ptr* gene may be involved in resistance mediated by other blast *R* genes. The fact that *Pi-ta* can recognize isolates with *AVR-Pita* (in O-137)^11,15^, and *Ptr* can recognize isolates with *AVR-Pita* haplotypes in US *M. oryzae* strains^12^ suggests that plants have developed a fail-safe mechanism to activate ETI (Fig. 3 and Supplementary Fig. 11). Continued investigation of underlying molecular mechanisms will help genetic engineering for improved blast resistance. We showed that destruction of *Ptr* in M2354 completely abolished the resistance mediated by *Pi-ta* and *Pi-ta2*^12^ (Supplementary Table 3 and Supplementary Data 1) suggesting that the integrity of the *Ptr* gene products are essential for transducing the pathogen signal. We attempted to address questions such as cellular location of *R* gene products and domains important for specificity of recognition. Despite that Ptr:GFP fusion protein was toxic to plant cells, we were able to demonstrate that differential localization occurred when two isomers of the susceptible *ptr* gene and the altered *ptr* gene product from M2354 were expressed suggesting that proper cytoplasmic and nuclear localization are important for their roles in signaling recognition and transduction.

We showed that the rice *Ptr* gene encodes an atypical resistance protein with an ARM repeat and that it is required for the function of a classical NLR resistance protein *Pi-ta*. An E3 ligase typified with both U-Box and ARM repeat domain has been shown to be involved in plant disease resistance^17,23^. ARM repeat proteins with diverse and fundamental functions have been found in many eukaryotes^23^. We speculated that Ptr with an ARM repeat domain and without a U-box could be a novel E3 ligase. However, we were unable to demonstrate E3 ligase activity in vitro suggesting that the Ptr protein is more likely involved in protein-protein interactions and least likely involved in protein degradation through ubiquitin pathway^17^. Noticeably, besides several other amino acid variations, there is a 3 bp deletion at the identical position as the 12 bp deletion in the *Ptr* allele of *Oryza longistaminata* (KN539074.1_FG004), which is one of six candidate genes for another broad-spectrum blast *R* gene named *Pi57(t)*^24^. A four amino acid deletion and nucleotides at positions 6925 and 6930 altering amino acids from M to K and E to K, respectively, were found to correlate with disease resistance specificity (Table 2 and Fig. 4a). We also found Katy *Ptr* type alleles with the deletion in the fourth exon in most of 48 rice varieties in the IRRI 3 K rice genome database (Supplementary Table 7 and Supplementary Data 2). Taken together, these data suggest that these 4 amino acid residues of the Ptr protein determine the specificity of pathogen recognition and transduction.

Plant breeders observed that many plant disease *R* genes are clustered within small genetic intervals. On rice chromosome 12, at least 19 blast *R* genes including *Pi-ta* and *Pi-ta2*/*Ptr* were mapped, and most of them are concentrated around the centromere region^24^. Rice cultivars with *Ptr*, some of them carrying *Pi-ta2* or both *Pi-ta* and *Pi-ta2*, suggest that *Pi-ta*, *Pi-ta2*, and *Ptr* genes have been introgressed into modern rice varieties independently by classical plant breeding^6,25^. However, we only identified 48 out of 2167 rice germplasm lines with *Ptr* based on IRRI 3 K database, 682 of them contain *Pi-ta* suggesting that *Ptr* has not been widely deployed (Fig. 5 and Supplementary Table 7). Because *Pi-ta* is physically close to *Ptr* on a chromosomal region lacking recombination, DNA markers for *Pi-ta*^26^ or for *Ptr* developed in this study can be excellent tools for improving blast resistance via marker-assisted breeding strategy.

The fact that most R proteins share a common structure is beneficial for engineering novel resistance specificities. However, much deeper studies of underlying mechanisms will be required before such a promise can be fulfilled. To date, most of the cloned plant *R* genes encode NLR proteins that act directly or indirectly to detect the fungal effectors for triggering disease resistance^1^. There are a few reports of non-NLR genes including a recessive *pi21* allele resulting in the loss of function of a proline-containing protein that confers durable blast resistance in rice^27^ and the *RPW8* gene for broad-spectrum resistance to downy mildew in *Arabidopsis*^28^. Additionally, a kinase-START gene was shown to confer resistance to a devastating disease caused by the stripe rust fungus *Puccinia striiformis*^29^, and a putative ABC transporter gene has been reported responsible for resistance to multiple fungal diseases in wheat^30^.

In summary, the cloning and characterization of *Ptr* reveals an atypical R protein. The discovery that a non-NLR gene *Ptr* is required together with an NLR gene *Pi-ta* for resistance to a wide range of blast isolates suggests that plants have evolved multifaceted mechanisms to cope with the rapid adaptation of plant pathogens^31^ (Supplementary Fig. 11). Stacking *Ptr* along with *Pi-ta* or other blast *R* genes near the centromere region of chromosome 12 may enhance the durability of rice disease resistance.

## Methods

### Plant materials and mapping populations used

Plant materials used in this study were mainly from the USDA National Plant Germplasm System (https://www.ars-grin.gov/npgs/). They are listed in Supplementary Table 9. Mapping populations used are: 1) An F_2_ population consisting of 11,618 individuals of the cross of the blast susceptible *indica* rice variety ‘Amane’ (PI 373335) from Sri Lanka with *Pi-ta*^32^ with the resistant *tropical japonica* variety ‘Katy’ with *Pi-ta*/*Pi-ta2*/*Ptr* (PI 527707); 2) An F_2:3_ population consisting of 300 individuals of the cross of a fast neutron induced Katy mutant M2354 with *Pi-ta*/*Pi-ta2* with Katy^11^; and 3) An F_2:3_ population consisting of 800 individuals of the cross of M2354 with Amane.

### Fungal material and disease evaluation

*M. oryzae* isolates used in this study were listed in the Supplementary Table 3 and Supplementary Data 1 and as indicated in Fig. 3^14^. Rice plants were grown in a greenhouse. Briefly, the germinated rice seeds were sowed in a black plastic insert (2.9 × 4.0 × 5.6 cm^3^) with sterilized local silt loam soils from the field. A tray containing 96 of these inserts was filled with water, which was then diffused into seedling via open holes at the bottom. The seedlings were grown in the greenhouse at 24 to 30 °C with an 8h dark and 16h light cycle until V3 to V4 stages^33^ and placed in a large black plastic bag for inoculation with a spore suspension of *M. oryzae*. Race (isolate) IB-49 (ML1) carrying *AVR-Pita1*^11^ was used to evaluate disease reactions of mapping populations. IB-49 (ML1) and IC-17 (ZN57) were used to inoculate CRISPR/Cas9 generated M_2_ plants. Spore production, suspension, inoculation, and evaluation were performed as described by Wang et al.^32^. Briefly, *M. oryzae* isolates were grown on oatmeal agar plates (BD Difco, NJ, USA) for 7 days at 21 to 24 °C under dark and white fluorescent light and were washed with 0.25% gelatin solution. Plants at 3- to 4- leaf stages^33^ were inoculated with 20 mL of the filtered spore suspensions (1 × 10^5^ spores/mL) with an airbrush and placed inside a sealed black plastic bag for 24 h at 21 to 24 °C. Then, inoculated plants were moved out from the bags and transferred to the cooling room with an 8h light and 16h dark cycle with 80% relative humidity for additional 6 days. Disease reactions were determined on the second youngest leaf, using a categorical rating system from 0 (resistant) to 5 (susceptible), in which 0 = no visible lesion, 1 = a few small point lesions, 2 = lesion size smaller than 2 mm without obvious fungal mass, 3 = 10% of leaf area with lesions bigger than 2 mm, 4 = > 10% and <50% of the leaf with lesions bigger than 3 mm, and 5 = > 50% of leaf area with lesions >3 mm^34^. The disease reactions were repeated three times and its mean value was used for analysis.

### Mapping and cloning of the *Ptr* gene

To map the *Ptr* gene, a bulk segregant analysis (BSA) was carried out using resistant (R) and susceptible (S) DNA pools, including 20 homozygous or heterozygous R and 20 homozygous S of the F_2_ derived from the cross of Amane with Katy, and phenotypes were confirmed in the F_3_ generation, respectively^35^. A total of 225 SSR markers distributing evenly on all 12 rice chromosomes were used to screen polymorphisms between R and S pools, and both parents. Five polymorphic SSR markers distinguishing R and S pools were identified. Linkage analysis was carried out using 162 individuals of F_2_ of the cross of Amane and Katy and the co-segregating and flanking markers in Fig. 1a were identified. To fine map *Ptr*, more SSR markers were developed or identified from the Gramene Markers Database (http://archive.gramene.org/markers/microsat/); InDel and CAPS markers were designed based on DNA variation within flanking markers in Fig. 1a between the *japonica* Nipponbare (http://rice.plantbiology.msu.edu/) and the *indica* IR64 (http://schatzlab.cshl.edu/data/rice/). Additionally, partial genomic sequences of the *Ptr* region of Katy and Amane were determined to identify more polymorphic markers. At the same time, an additional F_2_ population of 10,000 individuals was used to identify more recombinants because of a linkage block^36^. To identify the *Ptr* candidate gene, the genomic DNA fragments of all annotated genes predicted by MSU version 7.0 (http://rice.plantbiology.msu.edu/) between Katy and M2354 in the *Ptr* region were sequenced and analyzed. Additionally, the *Ptr* candidate gene in Amane and *Pi-ta*, *Pi-ta2* differential rice varieties were also sequenced. All PCR primers and markers were designed using Primer Premier 5 software and were listed in the Supplementary Table 10.

### RNA isolation and quantitative RT-PCR

Total RNA was extracted from various rice tissues using the RNeasy Plant Mini Kit (Qiagen, MD, USA) and treated with RNase-Free DNase (Qiagen). First-strand cDNA was synthesized using a PrimerScript II 1st Strand cDNA Synthesis Kit (Takara, CA, USA) with an oligo(dT) 18 primer according to the manufacture’s protocol. The qRT-PCR amplification was performed using SYBR® Premix Ex Taq^TM^ II (Takara) with Roche LightCycler® 96 System (CT, USA) following the manufacturer’s instruction. The qRT-PCR amplification was performed with three biological replicates, and the rice *Actin1* gene (LOC_Os03g50885) was used as an internal control for gene expression^37^.

### Subcellular localization assays

To determine subcellular localization of Ptr, two predicted Ptr isoforms, A and B, corresponding to the entire protein coding region (2592 bp and 2715 bp, without stop codon) were amplified from cDNA of seedling leaves of Katy, Amane, and M2354, and cloned into *Eco*RI and *Sal*I sites of the pYBA1132 vector to generate Ptr:EGFP, respectively. The fusion construct was transformed or co-transformed into rice protoplasts prepared from Nipponbare seedlings and *Nicotiana* using PEG-mediated or *Agrobacterium*-mediated methods as described^38^. The transformed protoplast cells were examined by a confocal laser-scanning microscope. The primers for localization study were listed in the Supplementary Table 11, and plasmid constructs were listed in the Supplementary Table 12.

### Targeted mutagenesis of *Ptr* in rice with CRISPR/Cas9

The *Ptr* gene in the Katy cultivar was targeted with two gRNA spacers that span 811 bp in the third exon of the gene. The highly specific gRNA spacer sequences (Supplementary Table 13) were designed using the CRISPR-plant database and website^39^. The corresponding spacer sequences were fused with transfer RNAs (tRNAs) using the golden gate assembly method^40^. The resulting polycistronic tRNA-gRNA gene was then introduced into *p*RGEB32 (Addgene #63142) by digesting the gene with *Fok*I and the plasmid with *Bsa*I to release compatible overhangs before ligation with T4 Ligase (New England Biolabs, MA, USA). The resulting binary vector (*p*KO-Ptr) was introduced via electroporation into the *Agrobacterium tumefaciens* strain EHA105. Rice calli derived from mature seeds of the cultivar Katy were transformed with *p*KO-Ptr using the standard protocol^40^. The transformation events were selected based on hygromycin B resistance and the regenerated plants were analyzed for genome-edited mutations in the target gene. Chromosomal deletions were detected by PCR with primers flanking the two target sites of each gene. InDels on the target sites with RE sites were detected by PCR-RE assay after PCR products encompassing the target were digested with the appropriate RE for 2 to 3 h. Selected PCR products from the transgenic CRISPR-edited lines were sequenced to determine the specific mutation. Double peaks were resolved with degenerate sequence decoding method^41^. The primers for CRISPR/Cas9 study were listed in the Supplementary Table 13.

### *M. oryzae* evaluation of CRISPR-edited transgenics

*M. oryzae* isolates IC-17 (ZN57) and IB-49 (ML1) were placed onto oatmeal agar (BD) plates and left to grow under constant white and UV light for eight to 9 days. Fungal spores from the agar were filtered with cheesecloth and collected into a 50 mL conical tube. Spore suspensions were measured with a hemocytometer and adjusted to a concentration of 1 × 10^5^ spores/mL for both the spray- and spot-inoculations. Two-week-old wild-type plants and mutant lines were spray-inoculated with spore suspension using an air brush via a diaphragm compressor (Whirlwind II Model 80–2, Badger Air-Brush Co., IL, USA). Inoculated plants were placed into 30”x12”x18” plastic bins containing 2 in of water to act as a dew chamber. After 24 h plants were removed from the plastic bins and placed in an environmental chamber at 28 °C for five additional days. Disease susceptibility or resistance was evaluated based on blast disease rating and lesion development as previously described in an earlier methods section (Fungal material and disease evaluation). Blast symptoms were imaged using a Nikon camera with 60 mm f/2.8D AF Micro-Nikon lens. Image J and Microsoft Excel were used to analyze data and generate graphs. For spot-inoculation, rice leaves from two- and four-week-old wild-type plants and mutant lines were cut and placed into plastic containers containing filter paper and water to maintain a moist environment for disease development. Twelve leaves were inoculated and tested for wild-type and each mutant line. After inoculation of two spots on each leaf with 10 uL of spore suspension, leaves were incubated at room temperature (22 °C) for 6 days. Blast symptoms were recorded with photography and analyzed with ImageJ software to calculate the lesion area.

### Ubiquitination assays

The entire coding region with 905 amino acids and partial coding sequence with 671 amino acids without predictive transmembrane domain of the N terminus of *Ptr* was cloned into *Sal*I and *Eco*RI sites of the pMAL-c5X vector (NEB) to generate in-frame fusion proteins, respectively. The proteins were expressed at 20 °C and purified using amylose attached magnetic beads with an affinity for MBP fusion proteins according to the manufacturer’s protocol (NEB). In vivo ubiquitination was performed as described^42^. To detect ubiquitination, the products were run through stain-free SDS-PAGE gels (Bio-Rad) and transferred onto Nitrocellulose membranes (Thermo Fisher Scientific, NC, USA). The membranes were blotted with anti-ubiquitin (Boston Biochem, MA, USA) and anti-MBP (NEB), respectively. The images were detected using ChemiDoc Imaging System (Bio-Rad, CA, USA). The primers and plasmid constructs for the ubiquitination assay study are listed in the Supplementary Tables 11 and 12, respectively.

### DNA sequence analysis

Because all functional polymorphism sites were identified in the fourth exon between Amane and Katy, we sequenced this exon to predict the origin and evolution of the *Ptr* gene. The fourth exon of the *Ptr* DNA sequences in various rice varieties was amplified using specific primers listed in the Supplementary Table 10. After gel purification, the DNA samples were sequenced at USDA-ARS Genomics and Bioinformatics Research Unit (Stoneville, Mississippi). The *Ptr* DNA sequences in *O. barthii* (OBART12G08790), *O. glaberrima* (ORGLA12G0083200), *O. meridionalis* (OMERI12G06260), *O. longistaminata* (KN539074.1_FG004), *O. rufipogon* (*ORUFI12G09760*), and *O. nivara* (ONIVA12G08670) were retrieved from the *Oryza* Map Alignment Project (OMAP)^21^ project on the Gramene website (http://www.gramene.org/). The DNA sequences were assembled using Vector NTI software and aligned with BioEdit version 7.2.5. A phylogenetic tree was constructed by the software MEGA 7.0. To determine the average number of nucleotide differences per site (Pi value) in *Ptr*, the entire coding region sequences of *Ptr* in Katy, YT14, YT16, Amane, Pi No.1, Pi No. 4, and BHA[43] were used to build the sliding window with DNasp v5.

### *Ptr* haplotype analysis in IRRI 3 K sequenced rice germplasm

To investigate the distribution of the resistant *Ptr* allele observed in Katy, we characterized haplotypes in the IRRI 3 K SNP Seek database^19,20^. The SNP data were extracted for the *Ptr* gene (LOC_Os12g18729) from the 4.8 M SNP database and haplotypes were characterized using 173 polymorphic sites within the exon regions of the gene. Data were obtained for 3,024 lines and lines that had missing data at SNP 10,833,409 were excluded, this SNP was correlated to the SNP InDel in sequence data presented in Supplementary Table 1. Lines with over 15% total missing data, with heterozygous alleles considered as missing, were excluded. Upon filtering, 2215 lines remained for haplotype characterization. Haplotypes were defined by having no SNP polymorphisms among lines in the same haplotype group. A total of 35 lines contained rare haplotypes (<10 lines within haplotype group) and these haplotype groups were not considered, and 13 lines were not able to be assigned to a haplotype group due to missing data. A total of 2167 lines were assigned to a haplotype group. The association of *Ptr* haplotypes to *Pi-ta* was conducted by comparing the functional *Pi-ta* SNP at 10,607,554 with the assigned *Ptr* haplotype group. Based on the sequence data, there appeared to be a single SNP difference between the haplotype groups that contained ‘Katy’ and ‘Drew’. These groups were not treated as separate haplotypes as it is known from pedigree that ‘Drew’ and ‘Katy’ contain the same allele through decent, as ‘Katy’ is the parent of ‘Drew’. SNPs of each *Ptr* haplotype of IRRI 3 K rice varieties in the coding regions were used to build the sliding window with DNasp v5 and a phylogenetic tree that was constructed with the software MEGA 7.0. See additional resource for varieties in each haplotype and predicted disease reaction and details of SNPs.

### Data availability

The full genomic sequence of the *Ptr* gene from Katy and Amane can be retrieved in GenBank (accession number: MG385185 and MG385186) (https://www.ncbi.nlm.nih.gov/search/?term=MG+385185). The coding region of rice varieties for sliding window in Fig. 5b can be retrieved from GenBank (accession number: MG385187 to MG385192) ((https://www.ncbi.nlm.nih.gov/search/?term=MG+385187). The fourth exon sequences for Fig. 5c can be retired from GenBank accession number: MG397008-MG397034 (https://www.ncbi.nlm.nih.gov/search/?term=MG+397008). The authors declare that all other data supporting the findings of this study are available within the manuscript and its supplementary files or are available from the corresponding author upon request.

## Electronic supplementary material

Supplementary Information

Description of Additional Supplementary Files

Supplementary Data 1

Supplementary Data 2
