# Supplementary material for: The rice blast resistance gene Ptr encodes an atypical protein required for broad-spectrum disease resistance
Source: Nat Commun. 2018 May 23;9:2039. doi: 10.1038/s41467-018-04369-4 (PMC5966436; doi:10.1038/s41467-018-04369-4)
Supplement: Supplementary file 1 — Supplementary Information [file 41467_2018_4369_MOESM1_ESM.pdf]

a

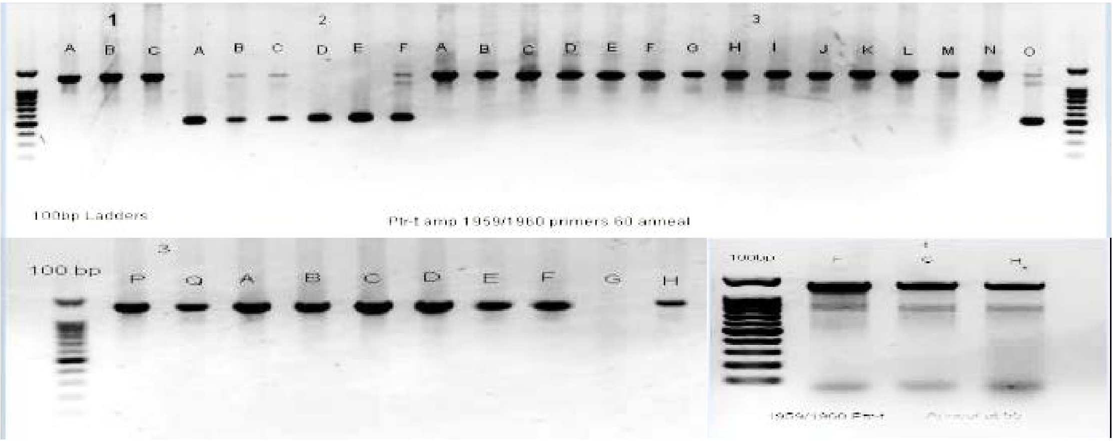

b

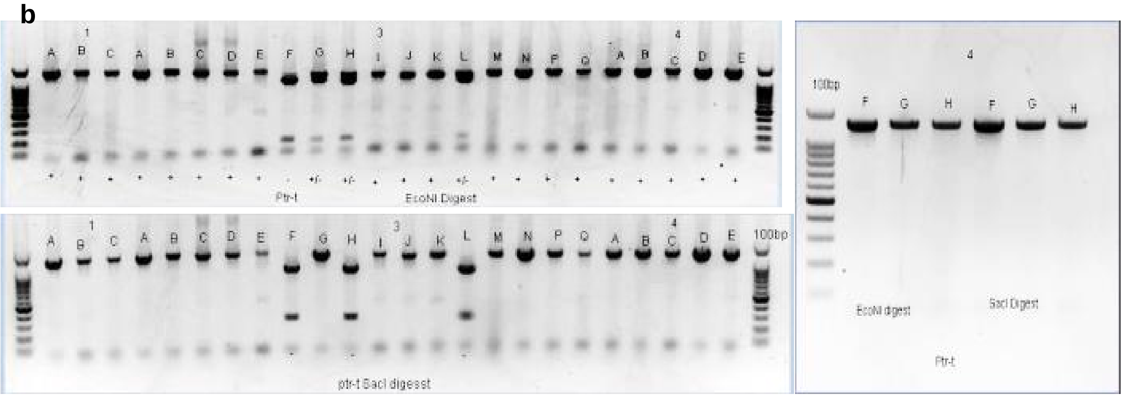

Supplementary Figure 1. Genotyping of indicated CRISPR-edited *ptr* mutant lines using PCR-RE assay.

(**a**) PCR amplification of the *Ptr* gene fragment with the PtrF/PtrR primers. (**b**) PCR-RE assay to detect insertion/deletions (InDels) in CRISPR-edited *ptr* mutant lines. *Eco*NI detects InDel at Protospacer 1 (PS1) target. *Sac*I detects InDel at Protospacer 2 (PS2) target. Note: Those amplified from true mutants edited by CRISPR should not be digested by *Eco*NI and/or *Sac*I. 3H was confirmed to be the same as the WT plant by DNA sequencing. The sequence of 3H is displayed in Figure 2 and shown to be identical

to wildtype (WT). The DNA fragments amplified from 3F,3H and 3L digested by *Eco*NI and *Sac*I suggesting that they contain the WT sequences and were discarded.

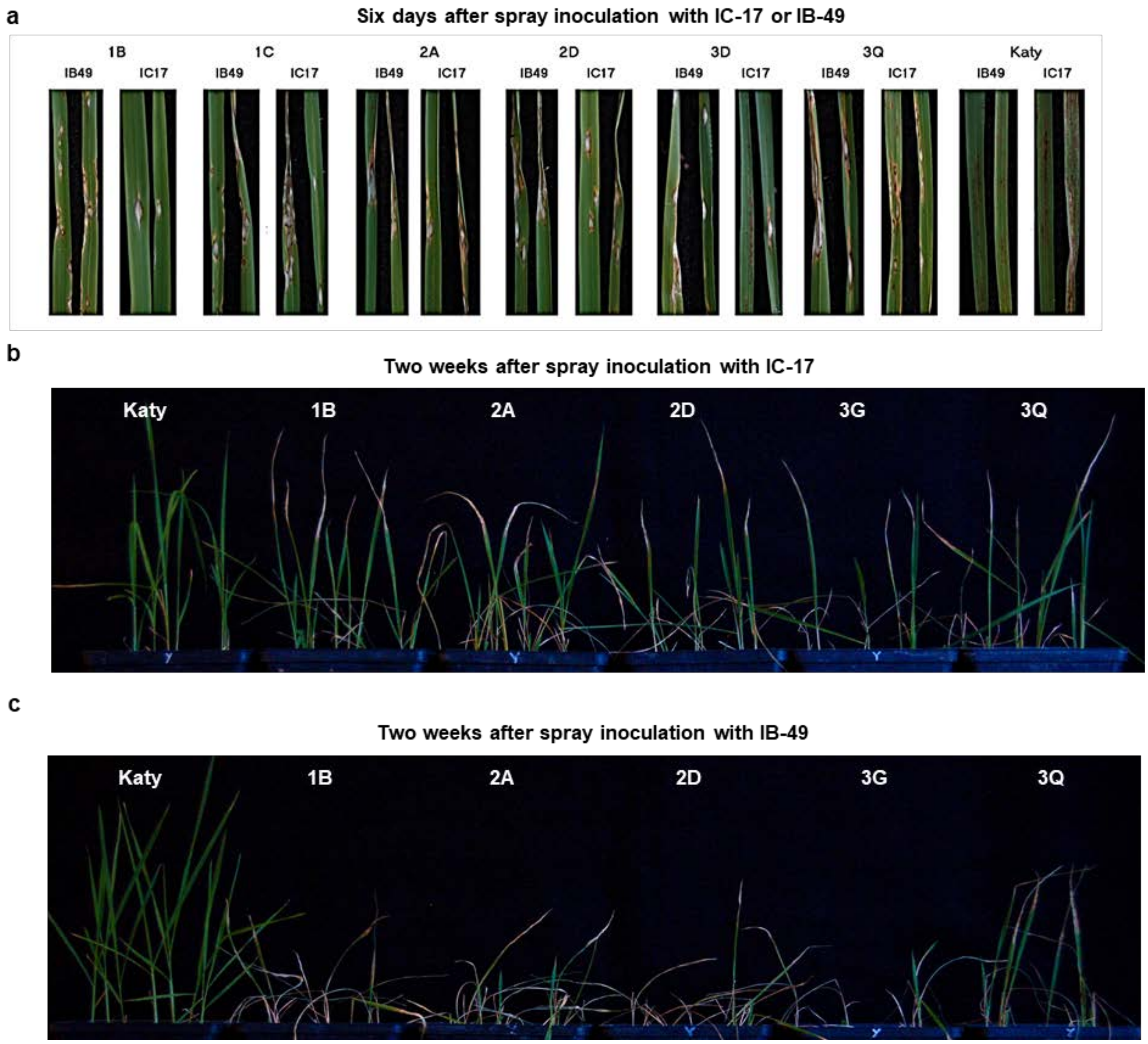

Supplementary Figure 2. Additional data on disease symptoms after spray-inoculation of *ptr* mutant lines.

(a) Lesion images from spray-inoculation with IC-17 and IB-49 pathotypes on WT Katy and CRISPR-edited *ptr* mutants at six days post-inoculation (DPI). **(b)** Image of WT Katy and CRISPR-edited *ptr* mutants at two weeks after inoculation with IC-17. **(c)** Image of WT Katy and CRISPR-edited *ptr* mutants at one week after inoculation with IB-49.

a

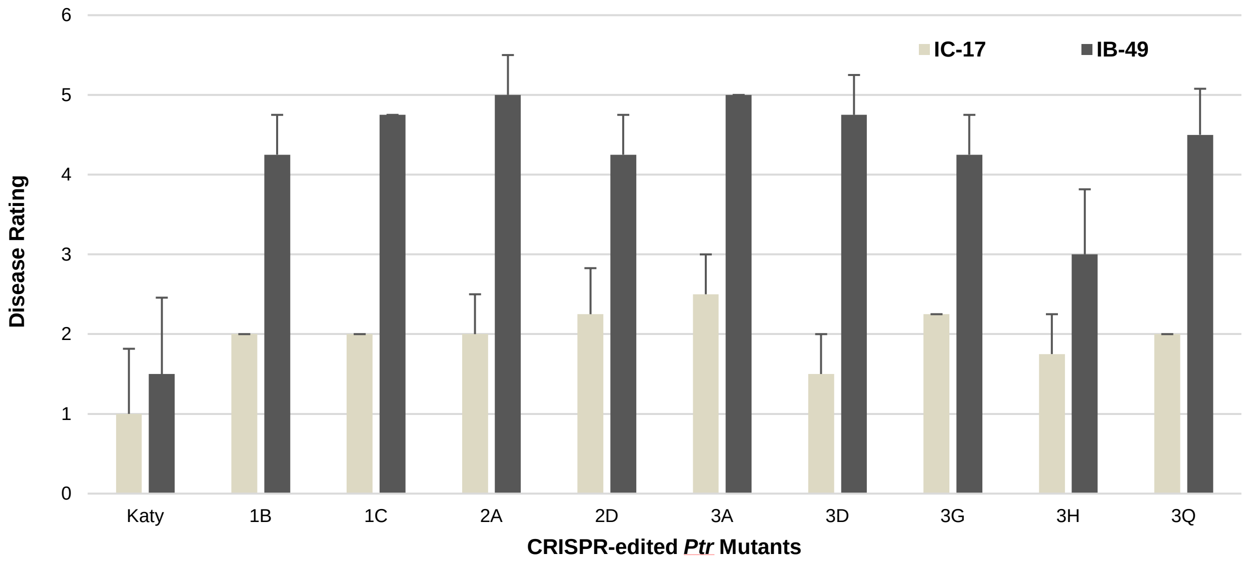

IC-17
IB-49
Disease Rating
0
1
2
3
4
5
6
Katy
1B
1C
2A
2D
3A
3D
3G
3H
3Q
CRISPR-edited Ptr Mutants

b

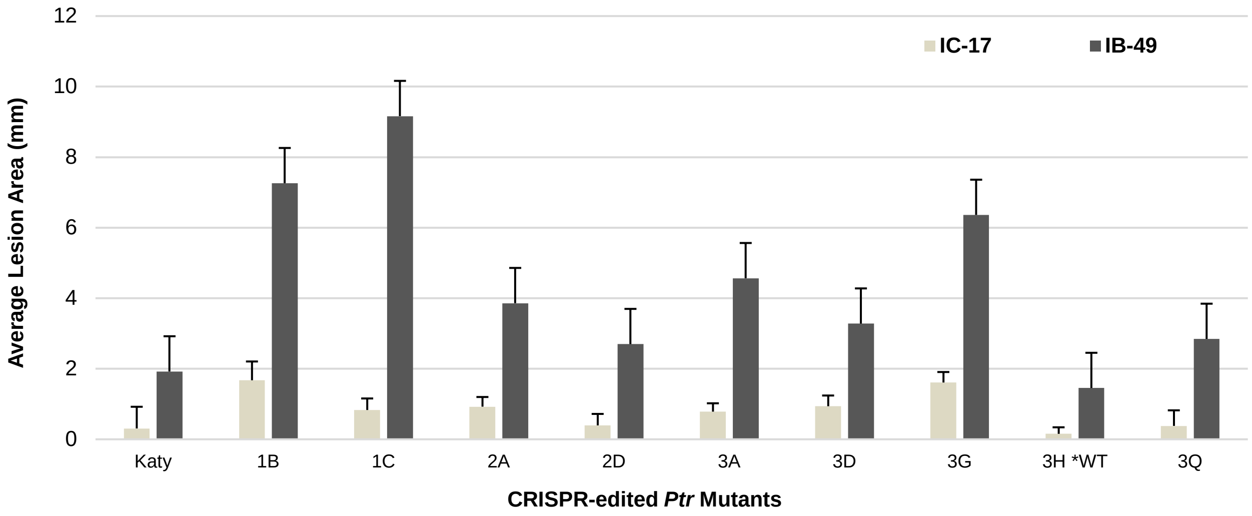

IC-17
IB-49
Average Lesion Area (mm)
0
2
4
6
8
10
12
Katy
1B
1C
2A
2D
3A
3D
3G
3H *WT
3Q
CRISPR-edited Ptr Mutants

Supplementary Figure 3. Disease evaluation based on spot-inoculation of indicated wildtype and *ptr* mutant leaves.

(**a**) Disease rating at six days after spot-inoculation of leaves from four-week-old plants. Disease rating was based on a scale of 1 to 5, 5 indicating the most severe lesion development. Data represent means ± s.d. (n=12). (**b**) Measurement of lesion area at six days after spot-inoculation of leaves from four-week-old plants. Error bars indicate standard deviation of average lesion area and disease rating. Average lesion area was determined by measuring the lesion area using ImageJ software.

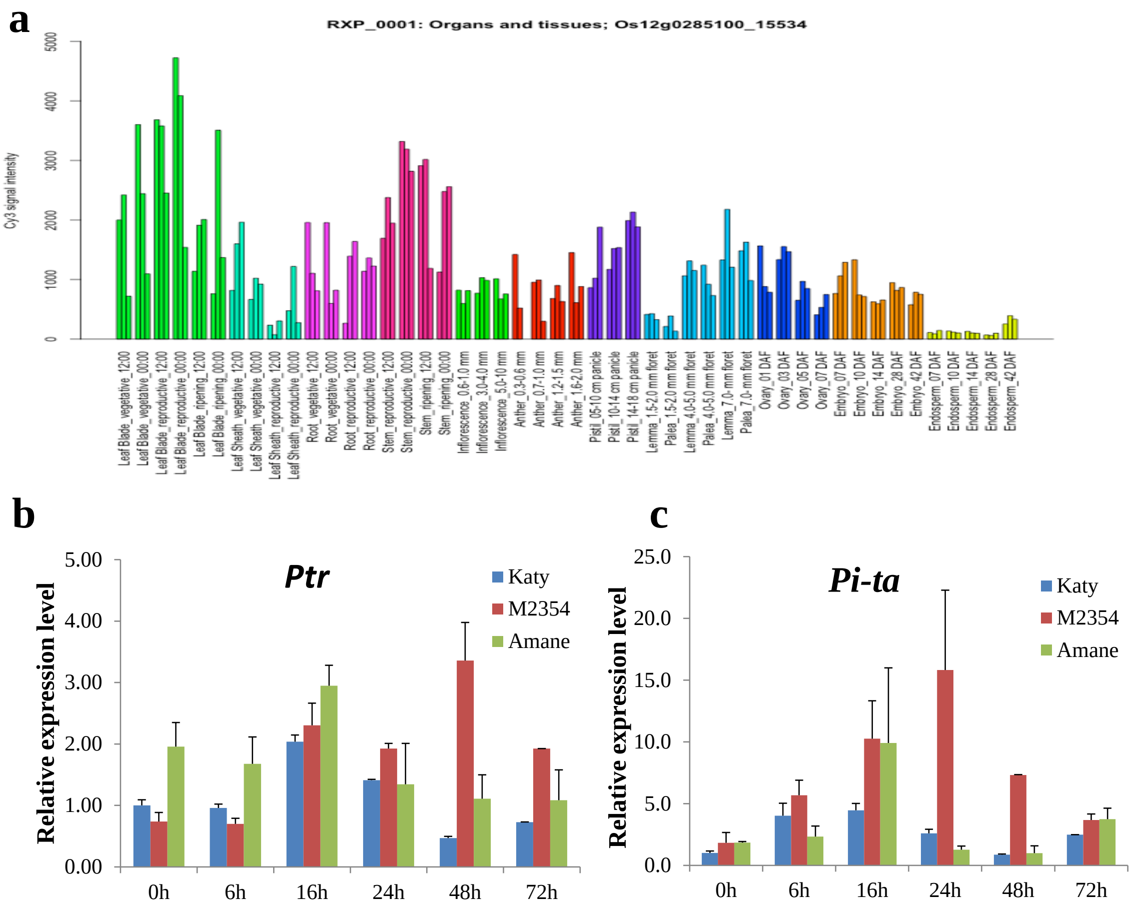

Supplementary Figure 4. Gene expression of *Ptr* and *Pi-ta* at different time points in both compatible and incompatible interactions.

(**a**) Constitutive expression of the *Ptr* gene in different parts of rice (data were retrieved from RiceXPro website-http://ricexpro.dna.affrc.go.jp/). (**b** and **c**) Profiles of gene expression of *Ptr* and *Pi-ta* in Katy, M2354, and Amane at different time points (0, 6, 16, 24, 48, 72 hours (h)) after inoculation with IB-49 (ML1) isolate detected by qRT-PCR using the relative $-2^{\triangle\triangle CT}$ method with *Actin1* as internal control, respectively. Data represent means with error bars showing ± s.d. (n=3).

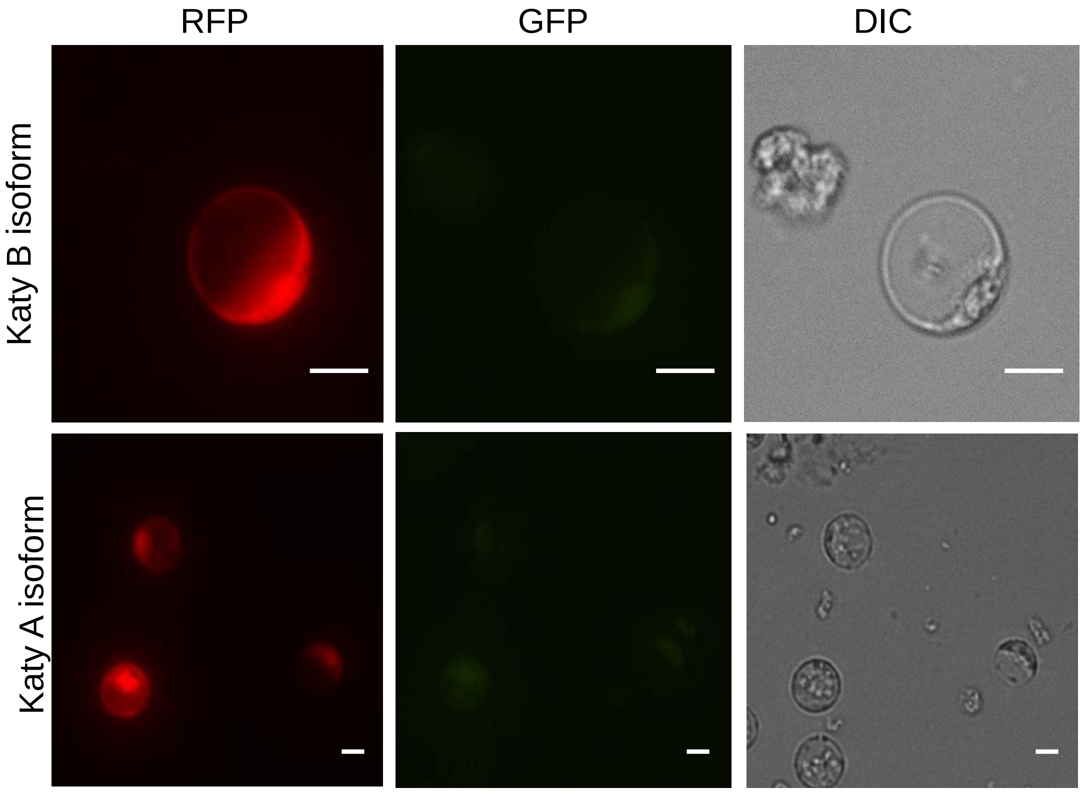

Supplementary Figure 5. Subcellular localization of two Ptr isoforms of Katy in rice protoplasts.

Green Fluorescent Protein (GFP)-fused Ptr proteins (two isoforms Katy-A and Katy-B) were co-expressed with Red Fluorescent Protein (RFP) in rice protoplasts. RFP was used as a marker for cytoplasm expression. Pictures were taken 16 hours after protoplast transfection. GFP, RFP, and differential interference contrast (DIC). Scale bars represent 10 μm.

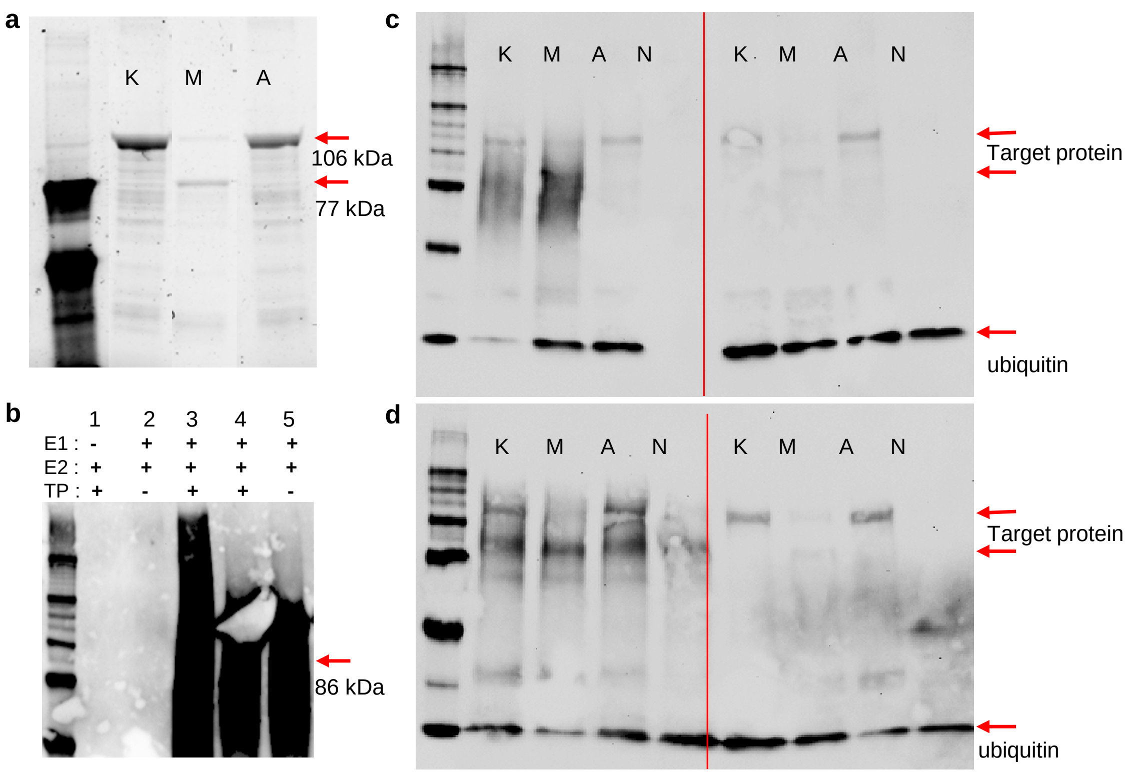

Supplementary Figure 6. Ubiquitination assay for Ptr.

Fusion proteins of Katy, M2354, and Amane. K, M, A, and N were assigned to Katy, M2354, Amane, and no protein added, respectively. (**a**) DNAs of Ptr in K, M, and A without first 235 amino acids (aa) from the start codon were ligated into pMAL to express the proteins. The fusion protein included a Maltose Binding Protein (MBP) tag (42.5 kDa). (**b**) Positive control for E3 assay and E2 ubiquitination. The first reaction contains a typical E3 ligase protein AvrPtoB (TP) and E2 protein SIUBC12without E1 (1); The second reaction contains E1 protein UBE1, SIUBC12 (E12), without AvrPtoB (TP) (2); The third reaction contains UBE1 (E1), SIUBC12 (E2), and AvrPtoB (TP) (GST+AvrPtoB is about 86 kDa) (3); The forth reaction contains Ptr protein of Katy (TP), UBE1 (E1), and E2 protein SIUBC15 (4); The fifth reaction contains only UBE1 (E1) and SIUBC15 (E2) as a negative control of the forth reaction (5). (**c**) Ubiquitination

assay with the E2 protein SlUBC15 and SlUBC9. SlUBC15 was applied to the left panel and SlUBC9 was applied to the right panel. (**d**) Ubiquitination assay with the E2 proteins SlUBC35 and SlUBC12. SlUBC35 was added to the left panel and SlUBC12 was added to the right panel. The same amount of UBE1 was added to each reaction.

```
                10        20        30        40        50        60        70        80        90       100
      ....|....|....|....|....|....|....|....|....|....|....|....|....|....|....|....|....|....|....|....|
Katy  ATGGATAGGCTCTGGGCGGCTCCTCCACTCTCCTCCCCTCTCTCATATCCAGTGGCTCGGGGTGGTGGGGCGTGGCCGACGCATCCGGCGGCGGAGCTCG
Pi1   ATGGATAGGCTCTGGGCGGCTCCTCCACTCTCCTCCCCTCTCTCATATCCAGTGGCTCGGGGTGGTGGGGCGTGGCCGACGCATCCGGCGGCGGAGCTCG
Pi4   ATGGATAGGCTCTGGGCGGCTCCTCCACTCTCCTCCCCTCTCTCATATCCAGTGGCTCGGGGTGGTGGGGCGTGGCCGACGCATCCGGCGGCGGAGCTCG
YT16  ATGGATAGGCTCTGGGCGGCTCCTCCACTCTCCTCCCCTCTCTCATATCCAGTGGCTCGGGGTGGTGGGGCGTGGCCGACGCATCCGGCGGCGGAGCTCG
Amane ATGGATAGGCTCTGGGCGGCTCCTCCACTCTCCTCCCCTCTCTCATATCCAGTGGCTCGGGGTGGTGGGGCGTGGCCGACGCATCCGGCGGCGGAGCTCG
BHA   ATGGATAGGCTCTGGGCGGCTCCTCCACTCTCCTCCCCTCTCTCATATCCAGTGGCTCGGGGTGGTGGGGCGTGGCCGACGCATCCGGCGGCGGAGCTCG

               110       120       130       140       150       160       170       180       190       200
      ....|....|....|....|....|....|....|....|....|....|....|....|....|....|....|....|....|....|....|....|
Katy  GGTGTTTAGCGGAGGAAGGATCGATGTCCGGCGCCGGACAGAGCCGTGGTCATCGCCTTGGATTACACATTGATTCGGATTGGCCAGAGGTCTTGTTGAT
Pi1   GGTGTTTAGCGGAGGAAGGATCGATGTCCGGCGCCGGACAGAGCCGTGGTCATCGCCTTGGATTACACATTGATTCGGATTGGCCAGAGGTCTTGTTGAT
Pi4   GGTGTTTAGCGGAGGAAGGATCGATGTCCGGCGCCGGACAGAGCCGTGGTCATCGCCTTGGATTACACATTGATTCGGATTGGCCAGAGGTCTTGTTGAT
YT16  GGTGTTTAGCGGAGGAAGGATCGATGTCCGGCGCCGGACAGAGCCGTGGTCATCGCCTTGGATTACACATTGATTCGGATTGGCCAGAGGTCTTGTTGAT
Amane GGTGTTTAGCGGAGGAAGGATCGATGTCCGGCGCCGGACAGAGCCGTGGTCATCGCCTTGGATTACACATTGATTCGGATTGGCCAGAGGTCTTGTTGAT
BHA   GGTGTTTAGCGGAGGAAGGATCGATGTCCGGCGCCGGACAGAGCCGTGGTCATCGCCTTGGATTACACATTGATTCGGATTGGCCAGAGGTCTTGTTGAT

               210       220       230       240       250       260       270       280       290       300
      ....|....|....|....|....|....|....|....|....|....|....|....|....|....|....|....|....|....|....|....|
Katy  CAATGACTATGCGGTGTTCATGGGGTACCTGTCGATGGTTGTCACCGGGACGGGGTTCCTGGTGCTCACGTGGTCCACCGTCATCCTCCTCGGTGGATTC
Pi1   CAATGACTATGCGGTGTTCATGGGGTACCTGTCGATGGTTGTCACCGGGACGGGGTTCCTGGTGCTCACGTGGTCCACCGTCATCCTCCTCGGTGGATTC
Pi4   CAATGACTATGCGGTGTTCATGGGGTACCTGTCGATGGTTGTCACCGGGACGGGGTTCCTGGTGCTCACGTGGTCCACCGTCATCCTCCTCGGTGGATTC
YT16  CAATGACTATGCGGTGTTCATGGGGTACCTGTCGATGGTTGTCACCGGGACGGGGTTCCTGGTGCTCACGTGGTCCACCGTCATCCTCCTCGGTGGATTC
Amane CAATGACTATGCGGTGTTCATGGGGTACCTGTCGATGGTTGTCACTGGGACGGGGTTCCTGGTGCTCACGTGGTCCACCGTCATCCTCCTCGGTGGATTC
BHA   CAATGACTATGCGGTGTTCATGGGGTACCTGTCGATGGTTGTCACCGGGACGGGGTTCCTGGTGCTCACGTGGTCCACCGTCATCCTCCTCGGTGGATTC

               310       320       330       340       350       360       370       380       390       400
      ....|....|....|....|....|....|....|....|....|....|....|....|....|....|....|....|....|....|....|....|
Katy  GTCTCCATGCTATCCAACAAGGACTTCTGGAGTCTCACGGTGATCACGCTCGTTCAAACAAGGATATTCGATGTTTTTCTGAATGGAAAAGTAAGCCACA
Pi1   GTCTCCATGCTATCCAACAAGGACTTCTGGAGTCTCACGGTGATCACGCTCGTTCAAACAAGGATATTCGATGTTTTTCTGAATGGAAAAGTAAGCCACA
Pi4   GTCTCCATGCTATCCAACAAGGACTTCTGGAGTCTCACGGTGATCACGCTCGTTCAAACAAGGATATTCGATGTTTTTCTGAATGGAAAAGTAAGCCACA
YT16  GTCTCCATGCTATCCAACAAGGACTTCTGGAGTCTCACGGTGATCACGCTCGTTCAAACAAGGATATTCGATGTTTTTCTGAATGGAAAAGTAAGCCACA
Amane GTCTCCATGCTATCCAACAAGGACTTCTGGAGTCTCACGGTGATCACGCTCGTTCAAACAAGGATATTCGATGTTTTTCTGAATGGAAAAGTAAGCCACA
BHA   GTCTCCATGCTATCCAACAAGGACTTCTGGAGTCTCACGGTGATCACGCTCGTTCAAACAAGGATATTCGATGTTTTTCTGAATGGAAAAGTAAGCCACA

               410       420       430       440       450       460       470       480       490       500
      ....|....|....|....|....|....|....|....|....|....|....|....|....|....|....|....|....|....|....|....|
Katy  TTGGGTACTCATTGAAGCGCTTGTGCAAGGCCGCACGCTTCATCGCGTTGCCCCATAACCATAAGAAGGTTGGGTTCAGGGGTGCTGTTCGAGTGCTTGT
Pi1   TTGGGTACTCATTGAAGCGCTTGTGCAAGGCCGCACGCTTCATCGCGTTGCCCCATAACCATAAGAAGGTTGGGTTCAGGGGTGCTGTTCGAGTGCTTGT
Pi4   TTGGGTACTCATTGAAGCGCTTGTGCAAGGCCGCACGCTTCATCGCGTTGCCCCATAACCATAAGAAGGTTGGGTTCAGGGGTGCTGTTCGAGTGCTTGT
YT16  TTGGGTACTCATTGAAGCGCTTGTGCAAGGCCGCACGCTTCATCGCGTTGCCCCATAACCATAAGAAGGTTGGGTTCAGGGGTGCTGTTCGAGTGCTTGT
Amane TTGGGTACTCATTGAAGCGCTTGTGCAAGGCCGCACGCTTCATCGCGTTGCCCCATAACCATAAGAAGGTTGGGTTCAGGGGTGCTGTTCGAGTGCTTGT
BHA   TTGGGTACTCATTGAAGCGCTTGTGCAAGGCCGCACGCTTCATCGCGTTGCCCCATAACCATAAGAAGGTTGGGTTCAGGGGTGCTGTTCGAGTGCTTGT

               510       520       530       540       550       560       570       580       590       600
      ....|....|....|....|....|....|....|....|....|....|....|....|....|....|....|....|....|....|....|....|
Katy  CTTCACCATCGTCTTGTGTCCGCTGTTCCTGCTCTACATGTTTGGGCTCTTCGTTTCCCCCTGGATTTCGCTGTGGCGTCTAATCCAGCAGGATTATGGC
Pi1   CTTCACCATCGTCTTGTGTCCGCTGTTCCTGCTCTACATGTTTGGGCTCTTCGTTTCCCCCTGGATTTCGCTGTGGCGTCTAATCCAGCAGGATTATGGC
Pi4   CTTCACCATCGTCTTGTGTCCGCTGTTCCTGCTCTACATGTTTGGGCTCTTCGTTTCCCCCTGGATTTCGCTGTGGCGTCTAATCCAGCAGGATTATGGC
YT16  CTTCACCATCGTCTTGTGTCCGCTGTTCCTGCTCTACATGTTTGGGCTCTTCGTTTCCCCCTGGATTTCGCTGTGGCGTCTAATCCAGCAGGATTACGGC
Amane CTTCACCATCGTCTTGTGTCCGCTGTTCCTGCTCTACATGTTTGGGCTCTTCGTTTCCCCCTGGATTTCGCTGTGGCGTCTAATCCAGCAGGATTACGGC
BHA   CTTCACCATCGTCTTGTGTCCGCTGTTCCTGCTCTACATGTTTGGGCTCTTCGTTTCCCCCTGGATTTCGCTGTGGCGTCTAATCCAGCAGGATTACGGC

               610       620       630       640       650       660       670       680       690       700
      ....|....|....|....|....|....|....|....|....|....|....|....|....|....|....|....|....|....|....|....|
Katy  GTGACGGCCGGAGACAGCAGCAGCAAGGCACACCTGCAGCCTGCGCTGGTGGTTCTCTACTCCCTGGCCCTGTTCCAGGGCGTCCTCTTCTACTACAGGG
Pi1   GTGACGGCCGGAGACAGCAGCAGCAAGGCACACCTGCAGCCTGCGCTGGTGGTTCTCTACTCCCTGGCCCTGTTCCAGGGCGTCCTCTTCTACTACAGGG
Pi4   GTGACGGCCGGAGACAGCAGCAGCAAGGCACACCTGCAGCCTGCGCTGGTGGTTCTCTACTCCCTGGCCCTGTTCCAGGGCGTCCTCTTCTACTACAGGG
YT16  GTGACGGCCGGAGACAGCAGCAGCAAGGCACACCTGCAGCCTGCGCTGGTGGTTCTCTACTCCCTGGCCCTGTTCCAGGGCGTCCTCTTCTACTACAGGG
Amane GTGACGGCCGGAGACAGCAGCAGCAAGGCACACCTGCAGCCTGCGCTGGTGGTTCTCTACTCCCTGGCCCTGTTCCAGGGCGTCCTCTTCTACTACAGGG
BHA   GTGACGGCCGGAGACAGCAGCAGCAAGGCACACCTGCAGCCTGCGCTGGTGGTTCTCTACTCCCTGGCCCTGTTCCAGGGCGTCCTCTTCTACTACAGGG

               710       720       730       740       750       760       770       780       790       800
      ....|....|....|....|....|....|....|....|....|....|....|....|....|....|....|....|....|....|....|....|
Katy  CCATCTCTGCTTGGGAAGAACAGAAGCTAGTGAAAGATGTGGCCGACAAATACATGTTTGATACAGTGTCGCGCAGTTCAGTTTCGGACTATTTACATGA
Pi1   CCATCTCTGCTTGGGAAGAACAGAAGCTAGTGAAAGATGTGGCCGACAAATACATGTTTGATACAGTGTCGCGCAGTTCAGTTTCGGACTATTTACATGA
Pi4   CCATCTCTGCTTGGGAAGAACAGAAGCTAGTGAAAGATGTGGCCGACAAATACATGTTTGATACAGTGTCGCGCAGTTCAGTTTCGGACTATTTACATGA
YT16  CCATCTCTGCTTGGGAAGAACAGAAGCTAGTGAAAGATGTGGCCGACAAATACATGTTTGATACAGTGTCGCGCAGTTCAGTTTCGGACTATTTACATGA
Amane CCATCTCTGCTTGGGAAGAACAGAAGCTAGTGAAAGATGTGGCCGACAAATACATGTTTGATACAGTGTCGCGCAGTTCAGTTTCGGACTATTTACATGA
BHA   CCATCTCTGCTTGGGAAGAACAGAAGCTAGTGAAAGATGTGGCCGACAAATACATGTTTGATACAGTGTCGCGCAGTTCAGTTTCGGACTATTTACATGA
```

```
              810       820       830       840       850       860       870       880       890       900
       ....|....|....|....|....|....|....|....|....|....|....|....|....|....|....|....|....|....|....|....|
Katy   GATCAAGGTGGGATGTGAGAATGACCCGTCCTTTGCCAGAGGGAGGAACCTGATCACATACGCTGTCAAGCTGATGGAATCCACATCACCGGATGGGTAC
Pi1    GATCAAGGTGGGATGTGAGAATGACCCGTCCTTTGCCAGAGGGAGGAACCTGATCACATACGCTGTCAAGCTGATGGAATCCACATCACCGGATGGGTAC
Pi4    GATCAAGGTGGGATGTGAGAATGACCCGTCCTTTGCCAGAGGGAGGAACCTGATCACATACGCTGTCAAGCTGATGGAATCCACATCACCGGATGGGTAC
YT16   GATCAAGGTCGGATGTGAGAATGACCCGTCCTTTGCCAGAGGGAGGAACCTGATCACATACGCTGTCAAGCTGATGGAATCCACATCACCGGATGGGTAC
Amane  GATCAAGGTGGGATGTGAGAATGACCCGTCCTTTGCCAGAGGGAGGAACCTGATCACATACGCTGTCAAGCTGATGGAATCCACATCACCGGATGGGTAC
BHA    GATCAAGGTCGGATGTGAGAATGACCCGTCCTTTGCCAGAGGGAGGAACCTGATCACATACGCTGTCAAGCTGATGGAATCCACATCACCGGATGGGTAC

              910       920       930       940       950       960       970       980       990      1000
       ....|....|....|....|....|....|....|....|....|....|....|....|....|....|....|....|....|....|....|....|
Katy   CTTTCAGGTGCACGGATTCTTGATACACTCATCAAGTTTAATAGAGATGATGCATCGGGGAGCGAATTACCGGGGCAGAGTATGCAGATATACAATATGA
Pi1    CTTTCAGGTGCACGGATTCTTGATACACTCATCAAGTTTAATAGAGATGATGCATCGGGGAGCGAATTACCGGGGCAGAGTATGCAGATATACAATATGA
Pi4    CTTTCAGGTGCACGGATTCTTGATACACTCATCAAGTTTAATAGAGATGATGCATCGGGGAGCGAATTACCGGGGCAGAGTATGCAGATATACAATATGA
YT16   CTTTCAGGTGCACGGATTCTTGATACACTCATCAAGTTTAATAGAGATGATGCATCGGGGAGCGAATTACCGGGGCAGAGTATGCAGATATACAATATGA
Amane  CTTTCAGGTGCACGGATTCTTGATACACTCATCAAGTTTAATAGAGATGATGCATCGGGGAGCGAATTACCGGGGCAGAGTATGCAGATATACAATATGA
BHA    CTTTCAGGTGCACGGATTCTTGATACACTCATCAAGTTTAATAGAGATGATGCATCGGGGAGCGAATTACCGGGGCAGAGTATGCAGATATACAATATGA

             1010      1020      1030      1040      1050      1060      1070      1080      1090      1100
       ....|....|....|....|....|....|....|....|....|....|....|....|....|....|....|....|....|....|....|....|
Katy   TTGGATCTGCATCCTCCAGTCCCATACTCCACAACTTAGTTCAGATGCTGGATTTCAAAAGTGCATATGATGGAGAGATCAGGTTGCGAGCCGCAAGGAT
Pi1    TTGGATCTGCATCCTCCAGTCCCATACTCCACAACTTAGTTCAGATGCTGGATTTCAAAAGTGCATATGATGGAGAGATCAGGTTGCGAGCCGCAAGGAT
Pi4    TTGGATCTGCATCCTCCAGTCCCATACTCCACAACTTAGTTCAGATGCTGGATTTCAAAAGTGCATATGATGGAGAGATCAGGTTGCGAGCCGCAAGGAT
YT16   TTGGATCTGCATCCTCCAGTCCCATACTCCACAACTTAGTTCAGATGCTGGATTTCAAAAGTGCATATGATGGAGAGATCAGGTTGCGAGCCGCAAGGAT
Amane  TTGGATCTGCATCCTCCAGTCCCATACTCCACAACTTAGTTCAGATGCTGGATTTCAAAAGTGCATATGATGGAGAGATCAGGTTGCGAGCCGCAAGGAT
BHA    TTGGATCTGCATCCTCCAGTCCCATACTCCACAACTTAGTTCAGATGCTGGATTTCAAAAGTGCATATGATGGAGAGATCAGGTTGCGAGCCGCAAGGAT

             1110      1120      1130      1140      1150      1160      1170      1180      1190      1200
       ....|....|....|....|....|....|....|....|....|....|....|....|....|....|....|....|....|....|....|....|
Katy   TGTTGAGCACTTTGCTGGTGAGGTCCGTTTAGACAAAATCCTGCAGGGGATTCGATGTGTATCTTCCTTGCTTGAACTCGAGCAGAAAGGATTTCAGAAT
Pi1    TGTTGAGCACTTTGCTGGTGAGGTCCGTTTAGACAAAATCCTGCAGGGGATTCGATGTGTATCTTCCTTGCTTGAACTCGAGCAGAAAGGATTTCAGAAT
Pi4    TGTTGAGCACTTTGCTGGTGAGGTCCGTTTAGACAAAATCCTGCAGGGGATTCGATGTGTATCTTCCTTGCTTGAACTCGAGCAGAAAGGATTTCAGAAT
YT16   TGTTGAGCACTTTGCTGGTGAGGTCCGTTTAGACAAAATCCTGCAGGGGATTCGATGTGTATCTTCCTTGCTTGAACTCGAGCAGAAAGGATTTCAGAAT
Amane  TGTTGAGCACTTTGCTGGTGAGGTCCGTTTAGACAAAATCCTGCAGGGGATTCGATGTGTATCTTCCTTGCTTGAACTCGAGCAGAAAGGATTTCAGAAT
BHA    TGTTGAGCACTTTGCTGGTGAGGTCCGTTTAGACAAAATCCTGCAGGGGATTCGATGTGTATCTTCCTTGCTTGAACTCGAGCAGAAAGGATTTCAGAAT

             1210      1220      1230      1240      1250      1260      1270      1280      1290      1300
       ....|....|....|....|....|....|....|....|....|....|....|....|....|....|....|....|....|....|....|....|
Katy   GACCACCATAGTTCTTTCCAAGAAGACGACGGCGACCAACTTTCTTTTGAAGAAGAGGATGATCACCAGATTTCTGTCAAAGAAAAGGATTATTACCCCA
Pi1    GACCACCATAGTTCTTTCCAAGAAGACGACGGCGACCAACTTTCTTTTGAAGAAGAGGATGATCACCAGATTTCTGTCAAAGAAAAGGATTATTACCCCA
Pi4    GACCACCATAGTTCTTTCCAAGAAGACGACGGCGACCAACTTTCTTTTGAAGAAGAGGATGATCACCAGATTTCTGTCAAAGAAAAGGATTATTACCCCA
YT16   GACCACCATAGTTCTTTCCAAGAAGACGACGGCGACCAACTTTCTTTTGAAGAAGAGGATGATCACCAGATTTCTGTCAAAGAAAAGGATTATTACCCCA
Amane  GACCACCATAGTTCTTTCCAAGAAGACGACGGCGACCAACTTTCTTTTGAAGAAGAGGATGATCACCAGATTTCTGTCAAAGAAAAGGATTATTACCCCA
BHA    GACCACCATAGTTCTTTCCAAGAAGACGACGGCGACCAACTTTCTTTTGAAGAAGAGGATGATCACCAGATTTCTGTCAAAGAAAAGGATTATTACCCCA

             1310      1320      1330      1340      1350      1360      1370      1380      1390      1400
       ....|....|....|....|....|....|....|....|....|....|....|....|....|....|....|....|....|....|....|....|
Katy   AAGATTATAAACAGATGCAACTTACAGGCATGCAGATCCTTTTAAAGCTCTCCTACGACAAGAACAACTTGTTCCTCATGAGCAACACAGATGATCCGGC
Pi1    AAGATTATAAACAGATGCAACTTACAGGCATGCAGATCCTTTTAAAGCTCTCCTACGACAAGAACAACTTGTTCCTCATGAGCAACACAGATGATCCGGC
Pi4    AAGATTATAAACAGATGCAACTTACAGGCATGCAGATCCTTTTAAAGCTCTCCTACGACAAGAACAACTTGTTCCTCATGAGCAACACAGATGATCCGGC
YT16   AAGATTATAAACAGATGCAACTTACAGGCATGCAGATCCTTTTAAAGCTCTCCTACGACAAGAACAACTTGTTCCTCATGAGCAACACAGATGATCCGGC
Amane  AAGATTATAAACAGATGCAACTTACAGGCATGCAGATCCTTTTAAAGCTCTCCTACGACAAGAACAACTTGTTCCTCATGAGCAACACAGATGATCCGGC
BHA    AAGATTATAAACAGATGCAACTTACAGGCATGCAGATCCTTTTAAAGCTCTCCTACGACAAGAACAACTTGTTCCTCATGAGCAACACAGATGATCCGGC

             1410      1420      1430      1440      1450      1460      1470      1480      1490      1500
       ....|....|....|....|....|....|....|....|....|....|....|....|....|....|....|....|....|....|....|....|
Katy   CTTGATCAACAAGATTGTGGCACTTATAACGTCCAAGGGATCACTTCACAAAAAACAACATAACGAATGGTCCTGTATGGCAGAGCTCGGGGTGAAGATA
Pi1    CTTGATCAACAAGATTGTGGCACTTATAACGTCCAAGGGATCACTTCACAAAAAACAACATAACGAATGGTCCTGTATGGCAGAGCTCGGGGTGAAGATA
Pi4    CTTGATCAACAAGATTGTGGCACTTATAACGTCCAAGGGATCACTTCACAAAAAACAACATAACGAATGGTCCTGTATGGCAGAGCTCGGGGTGAAGATA
YT16   CTTGATCAACAAGATTGTGGCACTTATAACGTCCAAGGGATCACTTCACAAAAAACAACATAACGAATGGTCCTGTATGGCAGAGCTCGGGGTGAAGATA
Amane  CTTGATCAACAAGATTGTGGCACTTATAACGTCCAAGGGATCACTTCACAAAAAACAACATAACGAATGGTCCTGTATGGCAGAGCTCGGGGTGAAGATA
BHA    CTTGATCAACAAGATTGTGGCACTTATAACGTCCAAGGGATCACTTCACAAAAAACAACATAACGAATGGTCCTGTATGGCAGAGCTCGGGGTGAAGATA

             1510      1520      1530      1540      1550      1560      1570      1580      1590      1600
       ....|....|....|....|....|....|....|....|....|....|....|....|....|....|....|....|....|....|....|....|
Katy   CTAAGCCGATTTATGCGATTTATGTATGGCCCTACAAAATCAAACAATATTCTGTGGCATGAAATATCAACAAGCAGCAAAGCAATCGGCACCTTGGAGA
Pi1    CTAAGCCGATTTATGCGATTTATGTATGGCCCTACAAAATCAAACAATATTCTGTGGCATGAAATATCAACAAGCAGCAAAGCAATCGGCACCTTGGAGA
Pi4    CTAAGCCGATTTATGCGATTTATGTATGGCCCTACAAAATCAAACAATATTCTGTGGCATGAAATATCAACAAGCAGCAAAGCAATCGGCACCTTGGAGA
YT16   CTAAGCCGATTTATGCGATTTATGTATGGCCCTACAAAATCAAACAATATTCTGTGGCATGAAATATCAACAAGCAGCAAAGCAATCGGCACCTTGGAGA
Amane  CTAAGCCGATTTATGCGATTTATGTATGGCCCTACAAAATCAAACAATATTCTGTGGCATGAAATATCAACAAGCAGCAAAGCAATCGGCACCTTGGAGA
BHA    CTAAGCCGATTTATGCGATTTATGTATGGCCCTACAAAATCAAACAATATTCTGTGGCATGAAATATCAACAAGCAGCAAAGCAATCGGCACCTTGGAGA
```

```
            1610      1620      1630      1640      1650      1660      1670      1680      1690      1700
      ....|....|....|....|....|....|....|....|....|....|....|....|....|....|....|....|....|....|....|....|
Katy  GTATTCTTGAGTGTGACCAATGTGACTCTGTGCTGAAGAAACACGCCATAAGGATTCTCAAAAGGATATTCATGGATACATCTTCGGCTATGGGTGAAGG
Pi1   GTATTCTTGAGTGTGACCAATGTGACTCTGTGCTGAAGAAACACGCCATAAGGATTCTCAAAAGGATATTCATGGATACATCTTCGGCTATGGGTGAAGG
Pi4   GTATTCTTGAGTGTGACCAATGTGACTCTGTGCTGAAGAAACACGCCATAAGGATTCTCAAAAGGATATTCATGGATACATCTTCGGCTATGGGTGAAGG
YT16  GTATTCTTGAGTGTGACCAATGTGACTCTGTGCTGAAGAAACACGCCATAAGGATTCTCAAAAGGATATTCATGGATACATCTTCGGCTATGGGTGAAGG
Amane GTATTCTTGAGTGTGACCAATGTGACTCTGTGCTGAAGAAACACGCCATAAGGATTCTCAAAAGGATATTCATGGATACATCTTCGGCTATGGGTGAAGG
BHA   GTATTCTTGAGTGTGACCAATGTGACTCTGTGCTGAAGAAACACGCCATAAGGATTCTCAAAAGGATATTCATGGATACATCTTCGGCTATGGGTGAAGG

            1710      1720      1730      1740      1750      1760      1770      1780      1790      1800
      ....|....|....|....|....|....|....|....|....|....|....|....|....|....|....|....|....|....|....|....|
Katy  AGACAGAGAAAGGTTCATTGGCTCCTTGATGGACATGTCTCTTCACAACAGTAATGGTGACTTTCAAAATCTGGCAGGTGTAGATCTGGCGCTCAAGAAA
Pi1   AGACAGAGAAAGGTTCATTGGCTCCTTGATGGACATGTCTCTTCACAACAGTAATGGTGACTTTCAAAATCTGGCAGGTGTAGATCTGGCGCTCAAGAAA
Pi4   AGACAGAGAAAGGTTCATTGGCTCCTTGATGGACATGTCTCTTCACAACAGTAATGGTGACTTTCAAAATCTGGCAGGTGTAGATCTGGCGCTCAAGAAA
YT16  AGACAGAGAAAGGTTCATTGGCTCCTTGATGGACATGTCTCTTCACAACAGTAATGGTGACTTTCAAAATCTGGCAGGTGTAGATCTGGCGCTCAAGAAA
Amane AGACAGAGAAAGGTTCATTGGCTCCTTGATGGACATGTCTCTTCACAACAGTAATGGTGACTTTCAAAATCTGGCAGGTGTAGATCTGGCGCTCAAGAAA
BHA   AGACAGAGAAAGGTTCATTGGCTCCTTGATGGACATGTCTCTTCACAACAGTAATGGTGACTTTCAAAATCTGGCAGGTGTAGATCTGGCGCTCAAGAAA

            1810      1820      1830      1840      1850      1860      1870      1880      1890      1900
      ....|....|....|....|....|....|....|....|....|....|....|....|....|....|....|....|....|....|....|....|
Katy  CAAGGGTTAAGCATTCTCAAAGAAATATACTTGAATCCATCTTCGATTATGGGCGAAGGAGACAGAGAAAGGTTCATCGGCTCCCTGATGGACATGTTTC
Pi1   CAAGGGTTAAGCATTCTCAAAGAAATATACTTGAATCCATCTTCGATTATGGGCGAAGGAGACAGAGAAAGGTTCATCGGCTCCCTGATGGACATGTTTC
Pi4   CAAGGGTTAAGCATTCTCAAAGAAATATACTTGAATCCATCTTCGATTATGGGCGAAGGAGACAGAGAAAGGTTCATCGGCTCCCTGATGGACATGTTTC
YT16  CAAGGGTTAAGCATTCTCAAAGAAATATACTTGAATCCATCTTCGATTATGGGCGAAGGAGACAGAGAAAGGTTCATCGGCTCCCTGATGGACATGTTTC
Amane CAAGGGTTAAGCATTCTCAAAGAAATATACTTGAATCCATCTTCGATTATGGGCGAAGGAGACAGAGAAAGGTTCATCGGCTCCCTGATGGACATGTTTC
BHA   CAAGGGTTAAGCATTCTCAAAGAAATATACTTGAATCCATCTTCGATTATGGGCGAAGGAGACAGAGAAAGGTTCATCGGCTCCCTGATGGACATGTTTC

            1910      1920      1930      1940      1950      1960      1970      1980      1990      2000
      ....|....|....|....|....|....|....|....|....|....|....|....|....|....|....|....|....|....|....|....|
Katy  TTGACAACAGTAAGGGCGACTTTGGAAATCTGCCAGGTGAAGATCTGGACCTCAAGAAACAAGAGTTAAGTATTCTCAAAGAAATATGCATGGATCCATC
Pi1   TTGACAACAGTAAGGGCGACTTTGGAAATCTGCCAGGTGAAGATCTGGACCTCAAGAAACAAGAGTTAAGTATTCTCAAAGAAATATGCATGGATCCATC
Pi4   TTGACAACAGTAAGGGCGACTTTGGAAATCTGCCAGGTGAAGATCTGGACCTCAAGAAACAAGAGTTAAGTATTCTCAAAGAAATATGCATGGATCCATC
YT16  TTGACAACAGTAAGGGCGACTTTGGAAATCTGCCAGGTGAAGATCTGGACCTCAAGAAACAAGAGTTAAGTATTCTCAAAGAAATATGCATGGATCCATC
Amane TTGACAACAGTAAGGGCGACTTTGGAAATCTGCCAGGTGAAGATCTGGACCTCAAGAAACAAGAGTTAAGTATTCTCAAAGAAATATGCATGGATCCATC
BHA   TTGACAACAGTAAGGGCGACTTTGGAAATCTGCCAGGTGAAGATCTGGACCTCAAGAAACAAGAGTTAAGTATTCTCAAAGAAATATGCATGGATCCATC

            2010      2020      2030      2040      2050      2060      2070      2080      2090      2100
      ....|....|....|....|....|....|....|....|....|....|....|....|....|....|....|....|....|....|....|....|
Katy  TTCGTTTATGGGCGAAGGTGACAGAGAAAAATTCATCGGCACCCTGATGGACATGTTTCTTCACAACAGCAAGGGCGACTTGTTTGAAAAACTAGCAGGT
Pi1   TTCGTTTATGGGCGAAGGTGACAGAGAAAAATTCATCGGCACCCTGATGGACATGTTTCTTCACAACAGCAAGGGCGACTTGTTTGAAAAACTAGCAGGT
Pi4   TTCGTTTATGGGCGAAGGTGACAGAGAAAAATTCATCGGCACCCTGATGGACATGTTTCTTCACAACAGCAAGGGCGACTTGTTTGAAAAACTAGCAGGT
YT16  TTCGTTTATGGGCGAAGGTGACAGAGAAAAATTCATCGGCACCCTGATGGACATGTTTCTTCACAACAGCAAGGGCGACTTGTTTGAAAAACTAGCAGGT
Amane TTCGTTTATGGGCGAAGGTGACAGAGAAAAATTCATCGGCACCCTGATGGACATGTTTCTTCACAACAGCAAGGGCGACTTGTTTGAAAAACTAGCAGGT
BHA   TTCGTTTATGGGCGAAGGTGACAGAGAAAAATTCATCGGCACCCTGATGGACATGTTTCTTCACAACAGCAAGGGCGACTTGTTTGAAAAACTAGCAGGT

            2110      2120      2130      2140      2150      2160      2170      2180      2190      2200
      ....|....|....|....|....|....|....|....|....|....|....|....|....|....|....|....|....|....|....|....|
Katy  GATGATCTGGTGCAGATATGTAGAAGAAGTGGAAGCAGTGCCACGATCATATTGAGGAAATATGGTCATGATATTGTTGATTGTATTGCTGATACTCGTT
Pi1   GATGATCTGGTGCAGATATGTAGAAGAAGTGGAAGCAGTGCCACGATCATATTGAGGAAATATGGTCATGATATTGTTGATTGTATTGCTGATACTCGTT
Pi4   GATGATCTGGTGCAGATATGTAGAAGAAGTGGAAGCAGTGCCACGATCATATTGAGGAAATATGGTCATGATATTGTTGATTGTATTGCTGATACTCGTT
YT16  GATGATCTGGTGCAGATATGTAGAAGAAGTGGAAGCAGTGCCACGATCATATTGAGGAAATATGGTCATGATATTGTTGATTGTATTGCTGATACTCGTT
Amane GATGATCTGGTGCAGATATGTAGAAGAAGTGGAAGCAGTGCCACGATCATATTGAGGAAATATGGTCATGATATTGTTGATTGTATTGCTGATACTCGTT
BHA   GATGATCTGGTGCAGATATGTAGAAGAAGTGGAAGCAGTGCCACGATCATATTGAGGAAATATGGTCATGATATTGTTGATTGTATTGCTGATACTCGTT

            2210      2220      2230      2240      2250      2260      2270      2280      2290      2300
      ....|....|....|....|....|....|....|....|....|....|....|....|....|....|....|....|....|....|....|....|
Katy  CAAGCGTATATAGCAGCATGCACAGAAAAATTGCAGCAAAGATCCTCAATCATCTGTGTAGTCCCTACTCCACTGACGAGGAACATCTTCAGAATCTGAA
Pi1   CAAGCGTATATAGCAGCATGCACAGAAAAATTGCAGCAAAGATCCTCAATCATCTGTGTAGTCCCTACTCCACTGACGAGGAACATCTTCAGAATCTGAA
Pi4   CAAGCGTATATAGCAGCATGCACAGAAAAATTGCAGCAAAGATCCTCAATCATCTGTGTAGTCCCTACTCCACTGACGAGGAACATCTTCAGAATCTGAA
YT16  CAAGCGTATATAGCAGCATGCACAGAAAAATTGCAGCAAAGATCCTCAATCATCTGTGTAGTCCCTACTCCACTGACGAGGAACATCTTCAGAATCTGAA
Amane CAAGCGTATATAGCAGCATGCACAGAAAAATTGCAGCAAAGATCCTCAATCATCTGTGTAGTCCCTACTCCACTGACGAGGAACATCTTCAGAATCTGAA
BHA   CAAGCGTATATAGCAGCATGCACAGAAAAATTGCAGCAAAGATCCTCAATCATCTGTGTAGTCCCTACTCCACTGACGAGGAACATCTTCAGAATCTGAA

            2310      2320      2330      2340      2350      2360      2370      2380      2390      2400
      ....|....|....|....|....|....|....|....|....|....|....|....|....|....|....|....|....|....|....|....|
Katy  GGAGGCCATCATTGATTTGATACCCAAGGTGCTAAGAGAAGCACTTGGTTGGGGGTTGACAGGAGAAGAGATACTGCGAGTGGCAGTTTCAGGCCTTGAA
Pi1   GGAGGCCATCATTGATTTGATACCCAAGGTGCTAAGAGAAGCACTTGGTTGGGGGTTGACAGGAGAAGAGATACTGCGAGTGGCAGTTTCAGGCCTTGAA
Pi4   GGAGGCCATCATTGATTTGATACCCAAGGTGCTAAGAGAAGCACTTGGTTGGGGGTTGACAGGAGAAGAGATACTGCGAGTGGCAGTTTCAGGCCTTGAA
YT16  GGAGGCCATCATTGATTTGATACCCAAGGTGCTAAGAGAAGCACTTGGTTGGGGGTTGACAGGAGAAGAGATACTGCGAGTGGCAGTTTCAGGCCTTGAA
Amane GGAGGCCATCATTGATTTGATACCCAAGGTGCTAAGAGAAGCACTTGGTTGGGGGTTGACAGGAGAAGAGATACTGCGAGTGGCAGTTTCAGGCCTTGAA
BHA   GGAGGCCATCATTGATTTGATACCCAAGGTGCTAAGAGAAGCACTTGGTTGGGGGTTGACAGGAGAAGAGATACTGCGAGTGGCAGTTTCAGGCCTTGAA
```

Supplementary Figure 7. Alignment of DNA sequences in the protein coding region of *Ptr* using representative varieties.

Katy and Pi4 contain *Pi-ta/Pi-ta2/Ptr*, Pi1 and Amane contain *Pi-ta*, and YT16 without

Katy alleles of *Pi-ta/ Pi-ta2/Ptr*. Black hull awned (BHA, MS-1996-9) is a weedy red

rice without the *Pi-ta* gene[43]. Differences in DNA are indicated by blue color. Genbank accession number: MG385187 to MG385192.

```
                              10        20        30        40        50        60        70        80        90
                      ....|....|....|....|....|....|....|....|....|....|....|....|....|....|....|....|....|....|
Oryza_rufipogon       GTGCTAAGAGAAGCACTTGGTTGGGGGTTGACAGGAAAAGAGATACAAGGAGTGGCAGTTTCAGGCCTTGAAGGTACCCAAGATGATGAC
Oryza_nivara          GTGCTAAGAGAAGCACTTGGTTGGGGGTTGACAGGAAAAGAGATACAAGGAGTGGCAGTTTCAGGCCTTGAAGGTACCCAAGATGATGAC
Oryza_meridionalis    GTGCTAAGAGAAGCACTTGGTTGGGGGTTGACAGGAGAAGAGATACTGCGAGTGGCAGTTTCAGGCCTTGAAGGTACCCAAGGTGATGAC
Oryza_longistaminata  GTGCTAAGAGAAGCACTTGGTTGGGGGTTGACAGGAAAAGAGATACAAGGAGTGGCAGTTTCAGGCCTTGAAGGTACCCAAGATGATGAC
Oryza_glaberrima      GTGCTAAGAGAAGCACTTGGTTGGGGGTTGACAGGAGAAGAGATACTGCGAGTGGCAGTTTCAGGCCTTGAAGGTACCCAAGATGATGAC
Oryza_barthii         GTGCTAAGAGAAGCACTTGGTTGGGGGTTGACAGGAGAAGAGATACTGCGAGTGGCAGTTTCAGGCCTTGAAGGTACCCAAGATGATGAC
Katy                  GTGCTAAGAGAAGCACTTGGTTGGGGGTTGACAGGAGAAGAGATACTGCGAGTGGCAGTTTCAGGCCTTGAAGGTACCCAAGATGATGAC
Sachiminori           GTGCTAAGAGAAGCACTTGGTTGGGGGTTGACAGGAGAAGAGATACTGCGAGTGGCAGTTTCAGGCCTTGAAGGTACCCAAGATGATGAC
Taducan               GTGCTAAGAGAAGCACTTGGTTGGGGGTTGACAGGAGAAGAGATACTGCGAGTGGCAGTTTCAGGCCTTGAAGGTACCCAAGATGATGAC
Pi No. 4              GTGCTAAGAGAAGCACTTGGTTGGGGGTTGACAGGAGAAGAGATACTGCGAGTGGCAGTTTCAGGCCTTGAAGGTACCCAAGATGATGAC
Pi No. 5              GTGCTAAGAGAAGCACTTGGTTGGGGGTTGACAGGAGAAGAGATACTGCGAGTGGCAGTTTCAGGCCTTGAAGGTACCCAAGATGATGAC
Reiho                 GTGCTAAGAGAAGCACTTGGTTGGGGGTTGACAGGAGAAGAGATACTGCGAGTGGCAGTTTCAGGCCTTGAAGGTACCCAAGATGATGAC
IRBLta2_Pi            GTGCTAAGAGAAGCACTTGGTTGGGGGTTGACAGGAGAAGAGATACTGCGAGTGGCAGTTTCAGGCCTTGAAGGTACCCAAGATGATGAC
IRBLta2_Re            GTGCTAAGAGAAGCACTTGGTTGGGGGTTGACAGGAGAAGAGATACTGCGAGTGGCAGTTTCAGGCCTTGAAGGTACCCAAGATGATGAC
Cybonnet              GTGCTAAGAGAAGCACTTGGTTGGGGGTTGACAGGAGAAGAGATACTGCGAGTGGCAGTTTCAGGCCTTGAAGGTACCCAAGATGATGAC
ADNY11                GTGCTAAGAGAAGCACTTGGTTGGGGGTTGACAGGAGAAGAGATACTGCGAGTGGCAGTTTCAGGCCTTGAAGGTACCCAAGATGATGAC
P3084F4-56-2-2        GTGCTAAGAGAAGCACTTGGTTGGGGGTTGACAGGAGAAGAGATACTGCGAGTGGCAGTTTCAGGCCTTGAAGGTACCCAAGATGATGAC
IR64                  GTGCTAAGAGAAGCACTTGGTTGGGGGTTGACAGGAGAAGAGATACTGCGAGTGGCAGTTTCAGGCCTTGAAGGTACCCAAGATGATGAC
Amane                 GTGCTAAGAGAAGCACTTGGTTGGGGGTTGACAGGAGAAGAGATACTGCGAGTGGCAGTTTCAGGCCTTGAAGGTACCCAAGATGATGAC
AR2001111             GTGCTAAGAGAAGCACTTGGTTGGGGGTTGACAGGAGAAGAGATACTGCGAGTGGCAGTTTCAGGCCTTGAAGGTACCCAAGATGATGAC
MO200213000           GTGCTAAGAGAAGCACTTGGTTGGGGGTTGACAGGAGAAGAGATACTGCGAGTGGCAGTTTCAGGCCTTGAAGGTACCCAAGATGATGAC
MS19968               GTGCTAAGAGAAGCACTTGGTTGGGGGTTGACAGGAGAAGAGATACTGCGAGTGGCAGTTTCAGGCCTTGAAGGTACCCAAGATGATGAC
AR-1994-10A           GTGCTAAGAGAAGCACTTGGTTGGGGGTTGACAGGAGAAGAGATACTGCGAGTGGCAGTTTCAGGCCTTGAAGGTACCCAAGATGATGAC
YT14                  GTGCTAAGAGAAGCACTTGGTTGGGGGTTGACAGGAGAAGAGATACTGCGAGTGGCAGTTTCAGGCCTTGAAGGTACCCAAGATGATGAC
Pi No. 2              GTGCTAAGAGAAGCACTTGGTTGGGGGTTGACAGGAGAAGAGATACTGCGAGTGGCAGTTTCAGGCCTTGAAGGTACCCAAGATGATGAC
Pi No. 1              GTGCTAAGAGAAGCACTTGGTTGGGGGTTGACAGGAGAAGAGATACTGCGAGTGGCAGTTTCAGGCCTTGAAGGTACCCAAGATGATGAC
Saber                 GTGCTAAGAGAAGCACTTGGTTGGGGGTTGACAGGAGAAGAGATACTGCGAGTGGCAGTTTCAGGCCTTGAAGGTACCCAAGATGATGAC
Yashiro-mochi         GTGCTAAGAGAAGCACTTGGTTGGGGGTTGACAGGAGAAGAGATACTGCGAGTGGCAGTTTCAGGCCTTGAAGGTACCCAAGATGATGAC
Kitaake               GTGCTAAGAGAAGCACTTGGTTGGGGGTTGACAGGAGAAGAGATACTGCGAGTGGCAGTTTCAGGCCTTGAAGGTACCCAAGATGATGAC
Nipponbare            GTGCTAAGAGAAGCACTTGGTTGGGGGTTGACAGGAGAAGAGATACTGCGAGTGGCAGTTTCAGGCCTTGAAGGTACCCAAGATGATGAC
YT16                  GTGCTAAGAGAAGCACTTGGTTGGGGGTTGACAGGAGAAGAGATACTGCGAGTGGCAGTTTCAGGCCTTGAAGGTACCCAAGATGATGAC
LTH                   GTGCTAAGAGAAGCACTTGGTTGGGGGTTGACAGGAAAAGAGATACAAGGAGTGGCAGTTTCAGGCCTTGAAGGTACCCAAGATGATGAC
IRBL9-W               GTGCTAAGAGAAGCACTTGGTTGGGGGTTGACAGGAAAAGAGATACAAGGAGTGGCAGTTTCAGGCCTTGAAGGTACCCAAGATGATGAC

                             100       110       120       130       140       150       160       170       180
                      ....|....|....|....|....|....|....|....|....|....|....|....|....|....|....|....|....|....|
Oryza_rufipogon       TGGAAATTGCAGGAAGCATTGGCGTCCCTCTGTGCAACCGTGTTTAACAGAATTGTCAGCAAGGATGCAGATTTGACTGCTCGGTTCAAC
Oryza_nivara          TGGAAATTGCAGGAAGCATTGGCGTCCCTCTGTGCAACCGTGTTTAACAGAATTGTCAGCAAGGATGCAGATTTGACTGCTCGGTTCAAC
Oryza_meridionalis    TGGAAATTGCAGGAAGCATTGGCGTCCCTCTGTGCAACCGTGTTTAACAGAATTATCAGCAAGGATGCAGATTTGACTGCTCGGTTCAAC
Oryza_longistaminata  TGGAAATTGCAGGAAGCATTGGCGTCCCTCTGTGCAACCGTGTTTAACAGAATTGTCAGCAAGGATGCAGATTTGACTGCTCGGTTCAAC
Oryza_glaberrima      TGGAAATTGCAGGAAGCATTGGCGTCCCTCTGTGCAACCGTGTTTAACAGAATTGTCAGCAAGGATGCAGATTTGACTGCTCGGTTCAAC
Oryza_barthii         TGGAAATTGCAGGAAGCATTGGCGTCCCTCTGTGCAACCGTGTTTAACAGAATTGTCAGCAAGGATGCAGATTTGACTGCTCGGTTCAAC
Katy                  TGGAAATTGCAGGAAGCATTGGCGTCCCTCTGTGCAACCGTGTTTAACAGAATTGTCAGCAAGGATGCAGATTTGACTGCTCGGTTCAAC
Sachiminori           TGGAAATTGCAGGAAGCATTGGCGTCCCTCTGTGCAACCGTGTTTAACAGAATTGTCAGCAAGGATGCAGATTTGACTGCTCGGTTCAAC
Taducan               TGGAAATTGCAGGAAGCATTGGCGTCCCTCTGTGCAACCGTGTTTAACAGAATTGTCAGCAAGGATGCAGATTTGACTGCTCGGTTCAAC
Pi No. 4              TGGAAATTGCAGGAAGCATTGGCGTCCCTCTGTGCAACCGTGTTTAACAGAATTGTCAGCAAGGATGCAGATTTGACTGCTCGGTTCAAC
Pi No. 5              TGGAAATTGCAGGAAGCATTGGCGTCCCTCTGTGCAACCGTGTTTAACAGAATTGTCAGCAAGGATGCAGATTTGACTGCTCGGTTCAAC
Reiho                 TGGAAATTGCAGGAAGCATTGGCGTCCCTCTGTGCAACCGTGTTTAACAGAATTGTCAGCAAGGATGCAGATTTGACTGCTCGGTTCAAC
IRBLta2_Pi            TGGAAATTGCAGGAAGCATTGGCGTCCCTCTGTGCAACCGTGTTTAACAGAATTGTCAGCAAGGATGCAGATTTGACTGCTCGGTTCAAC
IRBLta2_Re            TGGAAATTGCAGGAAGCATTGGCGTCCCTCTGTGCAACCGTGTTTAACAGAATTGTCAGCAAGGATGCAGATTTGACTGCTCGGTTCAAC
Cybonnet              TGGAAATTGCAGGAAGCATTGGCGTCCCTCTGTGCAACCGTGTTTAACAGAATTGTCAGCAAGGATGCAGATTTGACTGCTCGGTTCAAC
ADNY11                TGGAAATTGCAGGAAGCATTGGCGTCCCTCTGTGCAACCGTGTTTAACAGAATTGTCAGCAAGGATGCAGATTTGACTGCTCGGTTCAAC
P3084F4-56-2-2        TGGAAATTGCAGGAAGCATTGGCGTCCCTCTGTGCAACCGTGTTTAACAGAATTGTCAGCAAGGATGCAGATTTGACTGCTCGGTTCAAC
IR64                  TGGAAATTGCAGGAAGCATTGGCGTCCCTCTGTGCAACCGTGTTTAACAGAATTGTCAGCAAGGATGCAGATTTGACTGCTCGGTTCAAC
Amane                 TGGAAATTGCAGGAAGCATTGGCGTCCCTCTGTGCAACCGTGTTTAACAGAATTGTCAGCAAGGATGCAGATTTGACTGCTCGGTTCAAC
AR2001111             TGGAAATTGCAGGAAGCATTGGCGTCCCTCTGTGCAACCGTGTTTAACAGAATTGTCAGCAAGGATGCAGATTTGACTGCTCGGTTCAAC
MO200213000           TGGAAATTGCAGGAAGCATTGGCGTCCCTCTGTGCAACCGTGTTTAACAGAATTGTCAGCAAGGATGCAGATTTGACTGCTCGGTTCAAC
MS19968               TGGAAATTGCAGGAAGCATTGGCGTCCCTCTGTGCAACCGTGTTTAACAGAATTGTCAGCAAGGATGCAGATTTGACTGCTCGGTTCAAC
AR-1994-10A           TGGAAATTGCAGGAAGCATTGGCGTCCCTCTGTGCAACCGTGTTTAACAGAATTGTCAGCAAGGATGCAGATTTGACTGCTCGGTTCAAC
YT14                  TGGAAATTGCAGGAAGCATTGGCGTCCCTCTGTGCAACCGTGTTTAACAGAATTGTCAGCAAGGATGCAGATTTGACTGCTCGGTTCAAC
Pi No. 2              TGGAAATTGCAGGAAGCATTGGCGTCCCTCTGTGCAACCGTGTTTAACAGAATTGTCAGCAAGGATGCAGATTTGACTGCTCGGTTCAAC
Pi No. 1              TGGAAATTGCAGGAAGCATTGGCGTCCCTCTGTGCAACCGTGTTTAACAGAATTGTCAGCAAGGATGCAGATTTGACTGCTCGGTTCAAC
Saber                 TGGAAATTGCAGGAAGCATTGGCGTCCCTCTGTGCAACCGTGTTTAACAGAATTGTCAGCAAGGATGCAGATTTGACTGCTCGGTTCAAC
Yashiro-mochi         TGGAAATTGCAGGAAGCATTGGCGTCCCTCTGTGCAACCGTGTTTAACAGAATTGTCAGCAAGGATGCAGATTTGACTGCTCGGTTCAAC
Kitaake               TGGAAATTGCAGGAAGCATTGGCGTCCCTCTGTGCAACCGTGTTTAACAGAATTGTCAGCAAGGATGCAGATTTGACTGCTCGGTTCAAC
Nipponbare            TGGAAATTGCAGGAAGCATTGGCGTCCCTCTGTGCAACCGTGTTTAACAGAATTGTCAGCAAGGATGCAGATTTGACTGCTCGGTTCAAC
YT16                  TGGAAATTGCAGGAAGCATTGGCGTCCCTCTGTGCAACCGTGTTTAACAGAATTGTCAGCAAGGATGCAGATTTGACTGCTCGGTTCAAC
LTH                   TGGAAATTGCAGGAAGCATTGGCGTCCCTCTGTGCAACCGTGTTTAACAGAATTGTCAGCAAGGATGCAGATTTGACTGCTCGGTTCAAC
IRBL9-W               TGGAAATTGCAGGAAGCATTGGCGTCCCTCTGTGCAACCGTGTTTAACAGAATTGTCAGCAAGGATGCAGATTTGACTGCTCGGTTCAAC

                             190       200       210       220       230       240       250       260       270
                      ....|....|....|....|....|....|....|....|....|....|....|....|....|....|....|....|....|....|
Oryza_rufipogon       AACATCGCCGCTGGTATCTGCGACCAGGCAGCGAAGCCTCGCGTGACCTTCGCAGACCTCATCAAAGAAGCTGTGAAAGTTTATCGCATG
Oryza_nivara          AACATCGCCGCTGGTATCTGCGACCAGGCAGCGAAGCCTCGCGTGACCTTCGCAGACCTCATCAAAGAAGCTGTGAAAGTTCATCGCATG
Oryza_meridionalis    GACATCGCCGCTGGTATCTGCGACCAGACAGCGAAGCCTCGCATGACCTTCGCAGACCTCATCAACGAAGCTGTGAAAGTTCATCGCATC
Oryza_longistaminata  AACATCGCCGCTGGTATCTGCGACCAGGCAGCGAAGCCTCGCGTGACCTTCGCAGACCTCATCAAAGAAGCTGTGAAAGTTCATCGCATG
Oryza_glaberrima      AACATCGCCGCTGGTATCTGCGACCAGACAACGAAGCCTCGCATGACCTTCGCAGACCTCATCAACGAAGCTGTAAAAGTTCATCGCATC
Oryza_barthii         AACATCGCCGCTGGTATCTGCGACCAGACAACGAAGCCTCGCATGACCTTCGCAGACCTCATCAACGAAGCGGTAAAAGTTCATCGCATC
Katy                  AACATCGCCGCTGGTATCTGCGACCAGACAACGAAGCCTCGCATGACCTTCGCAGACCTCATCAACGAAGCTGTAAAAGTTCATCGCATC
Sachiminori           AACATCGCCGCTGGTATCTGCGACCAGACAACGAAGCCTCGCATGACCTTCGCAGACCTCATCAACGAAGCTGTAAAAGTTCATCGCATC
Taducan               AACATCGCCGCTGGTATCTGCGACCAGACAACGAAGCCTCGCATGACCTTCGCAGACCTCATCAACGAAGCTGTAAAAGTTCATCGCATC
Pi No. 4              AACATCGCCGCTGGTATCTGCGACCAGACAACGAAGCCTCGCATGACCTTCGCAGACCTCATCAACGAAGCTGTAAAAGTTCATCGCATC
Pi No. 5              AACATCGCCGCTGGTATCTGCGACCAGACAACGAAGCCTCGCATGACCTTCGCAGACCTCATCAACGAAGCTGTAAAAGTTCATCGCATC
Reiho                 AACATCGCCGCTGGTATCTGCGACCAGACAACGAAGCCTCGCATGACCTTCGCAGACCTCATCAACGAAGCTGTAAAAGTTCATCGCATC
IRBLta2_Pi            AACATCGCCGCTGGTATCTGCGACCAGACAACGAAGCCTCGCATGACCTTCGCAGACCTCATCAACGAAGCTGTAAAAGTTCATCGCATC
IRBLta2_Re            AACATCGCCGCTGGTATCTGCGACCAGACAACGAAGCCTCGCATGACCTTCGCAGACCTCATCAACGAAGCTGTAAAAGTTCATCGCATC
Cybonnet              AACATCGCCGCTGGTATCTGCGACCAGACAACGAAGCCTCGCATGACCTTCGCAGACCTCATCAACGAAGCTGTAAAAGTTCATCGCATC
ADNY11                AACATCGCCGCTGGTATCTGCGACCAGACAACGAAGCCTCGCATGACCTTCGCAGACCTCATCAACGAAGCTGTAAAAGTTCATCGCATC
P3084F4-56-2-2        AACATCGCCGCTGGTATCTGCGACCAGACAACGAAGCCTCGCATGACCTTCGCAGACCTCATCAACGAAGCTGTAAAAGTTCATCGCATC
```

```
IR64                  AACATCGCCGCTGGTATCTGCGACCAGACAACGAAGCCTCGCATGACCTTCGCAGACCTCATCAACGAAGCTGTAAAAGTTCATCGCATC
Amane                 AACATCGCCGCTGGTATCTGCGACCAGACAACGAAGCCTCGCATGACCTTCGCAGACCTCATCAACGAAGCTGTAAAAGTTCATCGCATC
AR2001111             AACATCGCCGCTGGTATCTGCGACCAGACAACGAAGCCTCGCATGACCTTCGCAGACCTCATCAACGAAGCTGTAAAAGTTCATCGCATC
MO200213000           AACATCGCCGCTGGTATCTGCGACCAGACAACGAAGCCTCGCATGACCTTCGCAGACCTCATCAACGAAGCTGTAAAAGTTCATCGCATC
MS19968               AACATCGCCGCTGGTATCTGCGACCAGACAACGAAGCCTCGCATGACCTTCGCAGACCTCATCAACGAAGCTGTAAAAGTTCATCGCATC
AR-1994-10A           AACATCGCCGCTGGTATCTGCGACCAGACAACGAAGCCTCGCATGACCTTCGCAGACCTCATCAACGAAGCTGTAAAAGTTCATCGCATC
YT14                  AACATCGCCGCTGGTATCTGCGACCAGACAACGAAGCCTCGCATGACCTTCGCAGACCTCATCAACGAAGCTGTAAAAGTTCATCGCATC
Pi No. 2              AACATCGCCGCTGGTATCTGCGACCAGACAACGAAGCCTCGCATGACCTTCGCAGACCTCATCAACGAAGCTGTAAAAGTTCATCGCATC
Pi No. 1              AACATCGCCGCTGGTATCTGCGACCAGACAACGAAGCCTCGCATGACCTTCGCAGACCTCATCAACGAAGCTGTAAAAGTTCATCGCATC
Saber                 AACATCGCCGCTGGTATCTGCGACCAGACAACGAAGCCTCGCATGACCTTCGCAGACCTCATCAATGAAGCTGTAAAAGTTCATCGCATC
Yashiro-mochi         AACATCGCCGCTGGTATCTGCGACCAGACAACGAAGCCTCGCATGACCTTCGCAGACCTCATCAACGAAGCTGTAAAAGTTCATCGCATC
Kitaake               AACATCGCCGCTGGTATCTGCGACCAGACAACGAAGCCTCGCATGACCTTCGCAGACCTCATCAATGAAGCTGTAAAAGTTCATCGCATC
Nipponbare            AACATCGCCGCTGGTATCTGCGACCAGACAACGAAGCCTCGCATGACCTTCGCAGACCTCATCAATGAAGCTGTAAAAGTTCATCGCATC
YT16                  AACATCGCCGCTGGTATCTGCGACCAGACAACGAAGCCTCGCATGACCTTCGCAGACCTCATCAATGAAGCTGTAAAAGTTCATCGCATC
LTH                   AACATCGCCGCTGGTATCTGCGACCAGGCAGCGA-GCCTCGCGTGACCTTCACAGACCTCATCAAAGAAGCTGTGAAAGTTCATCGCATG
IRBL9-W               AACATCGCCGCTGGTATCTGCGACCAGGCAGCGA-GCCTCGCGTGACCTTCACAGACCTCATCAAAGAAGCTGTGAAAGTTCATCGCATG

                              280       290       300       310       320       330       340       350       360
                      ....|....|....|....|....|....|....|....|....|....|....|....|....|....|....|....|....|....|
Oryza_rufipogon       GAATTTAAGAAACTAGAAGAACCAAAACCAGCTGCCAGCCCGGCACTTTACGAGTTTATGCCTGCATACTACCCCCGCCCTACTTTATG
Oryza_nivara          GAATTTAAGAAACTAGAAGAACAAAAACCAGCTGCCAGCCCGGCACTTTACGAGTTTATGCCTGCATACTACCCCCGCCCCACTTTATG
Oryza_meridionalis    GAATTTAAAAAACCAGAAAAACCAAAACCAGCTGCCAGGCCGGAACTGTACGAGTTTATGCCTGCGTACTACCCCCGCCCCACTTTATG
Oryza_longistaminata  GAATTTAAGAAACCAGAA---CCAAAACCAGCTGCCAGCCCGGCACTTTACGAGTTTATGCCTGCGTACTACCCCCGCCCCACTTTATG
Oryza_glaberrima      GAATTTAAAAAACCAGAAAAACCAAAACCAGCTGCCAGGCCGGAACTGTACGAGTTTATGCCTGCGAAATACCCCCGCCCCACTTTATG
Oryza_barthii         GAATTTAAAAAACCAGAAATACCAAAACCAGCTGCCAAGCCGGAACTGTACGAGTTTATGCCTGCGAAATACCCCCGCCCCACTTTATG
Katy                  GAATTTATG------------CCTGAACCACCTGCCAAGCCGGAACCGTACGAGTTTATGCCTGCGAAATACCCCCCGCCCCACTATATG
Sachiminori           GAATTTATG------------CCTGAACCACCTGCCAAGCCGGAACCGTACGAGTTTATGCCTGCGAAATACCCCCCGCCCCACTATATG
Taducan               GAATTTATG------------CCTGAACCACCTGCCAAGCCGGAACCGTACGAGTTTATGCCTGCGAAATACCCCCCGCCCCACTATATG
Pi No. 4              GAATTTATG------------CCTGAACCACCTGCCAAGCCGGAACCGTACGAGTTTATGCCTGCGAAATACCCCCCGCCCCACTATATG
Pi No. 5              GAATTTATG------------CCTGAACCACCTGCCAAGCCGGAACCGTACGAGTTTATGCCTGCGAAATACCCCCCGCCCCACTATATG
Reiho                 GAATTTATG------------CCTGAACCACCTGCCAAGCCGGAACCGTACGAGTTTATGCCTGCGAAATACCCCCCGCCCCACTATATG
IRBLta2_Pi            GAATTTATG------------CCTGAACCACCTGCCAAGCCGGAACCGTACGAGTTTATGCCTGCGAAATACCCCCCGCCCCACTATATG
IRBLta2_Re            GAATTTATG------------CCTGAACCACCTGCCAAGCCGGAACCGTACGAGTTTATGCCTGCGAAATACCCCCCGCCCCACTATATG
Cybonnet              GAATTTATG------------CCTGAACCACCTGCCAAGCCGGAACCGTACGAGTTTATGCCTGCGAAATACCCCCCGCCCCACTATATG
ADNY11                GAATTTATG------------CCTGAACCACCTGCCAAGCCGGAACCGTACGAGTTTATGCCTGCGAAATACCCCCCGCCCCACTATATG
P3084F4-56-2-2        GAATTTATG------------CCTGAACCACCTGCCAAGCCGGAACCGTACGAGTTTATGCCTGCGAAATACCCCCCGCCCCACTATATG
IR64                  GAATTTATG------------CCTGAACCACCTGCCAAGCCGGAACCGTACGAGTTTATGCCTGCGAAATACCCCCCGCCCCACTATATG
Amane                 GAATTTAAAAAACCAGAAAAACCAAAACCAGCTGCCAGGCCGGAACTGTACGAGTTTATGCCTGCGAAATACCCCCCGCCCCACTATATG
AR2001111             GAATTTAAAAAACCAGAAAAACCAAAACCACCTGCCAAGCCGGAACTGTACGAGTTTATGCCTGCGAAATACCCCCCGCCCCACTATATG
MO200213000           GAATTTAAAAAACCAGAAAAACCAAAACCACCTGCCAAGCCGGAACTGTACGAGTTTATGCCTGCGAAATACCCCCCGCCCCACTATATG
MS19968               GAATTTAAAAAACCAGAAAAACCAAAACCACCTGCCAAGCCGGAACTGTACGAGTTTATGCCTGCGAAATACCCCCCGCCCCACTATATG
AR-1994-10A           GAATTTAAAAAACCAGAAAAACAAGAACCAGCTGCCAGGCCGGAACTGTACGAGTTTGTGACTGCGAAATACACCCCGCCCCACTTTATG
YT14                  GAATTTAAAAAACCAGAAAAACCAAAACCACCTGCCAAGCCGGAACCGTACGAGTTTATGCCTGCGAAATACCCCCCGCCCCACTATATG
Pi No. 2              GAATTTAAAAAACCAGAAAAACCAAAACCACCTGCCAAGCCGGAACCGTACGAGTTTATGCCTGCGAAATACCCCCCGCCCCACTATATG
Pi No. 1              GAATTTAAAAAACCAGAAAAACCAAAACCACCTGCCAAGCCGGAACCGTACGAGTTTATGCCTGCGAAATACCCCCCGCCCCACTATATG
Saber                 GAATTTAAAAAACCAGAAAAACCAAAACCAGCTGCCAGGCCGGAACTGTACGAGTTTGTGACTGCGAAATACACCCCGCCCCACTTTATG
Yashiro-mochi         GAATTTAAAAAACCAGAAAAACCAAAACCACCTGCCAAGCCGGAACCGTACGAGTTTATGCCTGCGAAATACCCCCCGCCCCACTATATG
Kitaake               GAATTTAAAAAACCAGAAAAACCAAAACCAGCTGCCAGGCCGGAACTGTACGAGTTTGTGCCTGCGAAATACACCCCGCCCCACTTTATG
Nipponbare            GAATTTAAAAAACCAGAAAAACCAAAACCAGCTGCCAGGCCGGAACTGTACGAGTTTGTGACTGCGAAATACACCCCGCCCCACTTTATG
YT16                  GAATTTAAAAAACCAGAAAAACCAAAACCAGCTGCCAGGCCGGAACTGTACGAGTTTGTGACTGCGAAATACACCCCGCCCCACTTTATG
LTH                   GAATTTAAGAAACCAGAAGAACCAAAACCAGCTGCCAGCCCGGCACTTTACGAGTTTATGCCTGCATACTACCCCCGCCCCACTTTATG
IRBL9-W               GAATTTAAGAAACCAGAAGAACCAAAACCAGCTGCCAGCCCGGCACTTTACGAGTTTATGCCTGCATACTACCCCCGCCCCACTTTATG

                              370       380       390       400
                      ....|....|....|....|....|....|....|....|..
Oryza_rufipogon       TATTTGAGCGAGGAAGATCCCAACGCGTGTTGTATCTCTTGA
Oryza_nivara          TATTTGAGCGAGGAAGATCCCAACGCGTGTTGTATCTCTTGA
Oryza_meridionalis    TATTTGGGCGAGGAAGATCCCAACGCGTGTTGTATCTCTTGA
Oryza_longistaminata  TATTTGAGCGAGGAAGATCCCAACGCGTGTTGTATCTCTTGA
Oryza_glaberrima      TATTTGGGCGAGGAAGATCCCAACGCGTGTTGTATCTCTTGA
Oryza_barthii         TATTTGGGCGAGGAAGATCCCAACGCGTGTTGTATCTCTTGA
Katy                  TTTTTGGTCGAGGAAGATCCCAACGCGTGTTGTATCTCTTGA
Sachiminori           TTTTTGGTCGAGGAAGATCCCAACGCGTGTTGTATCTCTTGA
Taducan               TTTTTGGTCGAGGAAGATCCCAACGCGTGTTGTATCTCTTGA
Pi No. 4              TTTTTGGTCGAGGAAGATCCCAACGCGTGTTGTATCTCTTGA
Pi No. 5              TTTTTGGTCGAGGAAGATCCCAACGCGTGTTGTATCTCTTGA
Reiho                 TTTTTGGTCGAGGAAGATCCCAACGCGTGTTGTATCTCTTGA
IRBLta2_Pi            TTTTTGGTCGAGGAAGATCCCAACGCGTGTTGTATCTCTTGA
IRBLta2_Re            TTTTTGGTCGAGGAAGATCCCAACGCGTGTTGTATCTCTTGA
Cybonnet              TTTTTGGTCGAGGAAGATCCCAACGCGTGTTGTATCTCTTGA
ADNY11                TTTTTGGTCGAGGAAGATCCCAACGCGTGTTGTATCTCTTGA
P3084F4-56-2-2        TTTTTGGTCGAGGAAGATCCCAACGCGTGTTGTATCTCTTGA
IR64                  TTTTTGGTCGAGGAAGATCCCAACGCGTGTTGTATCTCTTGA
Amane                 TTTTTGGTCGAGGAAGATCCCAACGCGTGTTGTATCTCTTGA
AR2001111             TTTTTGGTCGAGGAAGATCCCAACGCGTGTTGTATCTCTTGA
MO200213000           TTTTTGGTCGAGGAAGATCCCAACGCGTGTTGTATCTCTTGA
MS19968               TTTTTGGTCGAGGAAGATCCCAACGCGTGTTGTATCTCTTGA
AR-1994-10A           TTTTTGGGCGAGGAAGATCCCAACGCGTGTTGTATCTCTTGA
YT14                  TTTTTGGTCGAGGAAGATCCCAACGCGTGTTGTATCTCTTGA
Pi No. 2              TTTTTGGTCGAGGAAGATCCCAACGCGTGTTGTATCTCTTGA
Pi No. 1              TTTTTGGTCGAGGAAGATCCCAACGCGTGTTGTATCTCTTGA
Saber                 TTTTTGGGCGAGGAAGATCCCAACGCGTGTTGTATCTCTTGA
Yashiro-mochi         TTTTTGGTCGAGGAAGATCCCAACGCGTGTTGTATCTCTTGA
Kitaake               TTTTTGGGCGAGGAAGATCCCAACGCGTGTTGTATCTCTTGA
Nipponbare            TTTTTGGGCGAGGAAGATCCCAACGCGTGTTGTATCTCTTGA
YT16                  TTTTTGGGCGAGGAAGATCCCAACGCGTGTTGTATCTCTTGA
LTH                   TATTTGAGCGAGGAAGATCCCAACGCGTGTTGTATCTCTTGA
IRBL9-W               TATTTGAGCGAGGAAGATCCCAACGCGTGTTGTATCTCTTGA
```

Supplementary Figure 8. DNA sequence alignment of the fourth exon of the protein coding region of *Ptr* in *Oryza species*.

The detailed information of rice varieties is listed in Supplementary Table 6. Differences in DNA sequences are indicated by blue color.

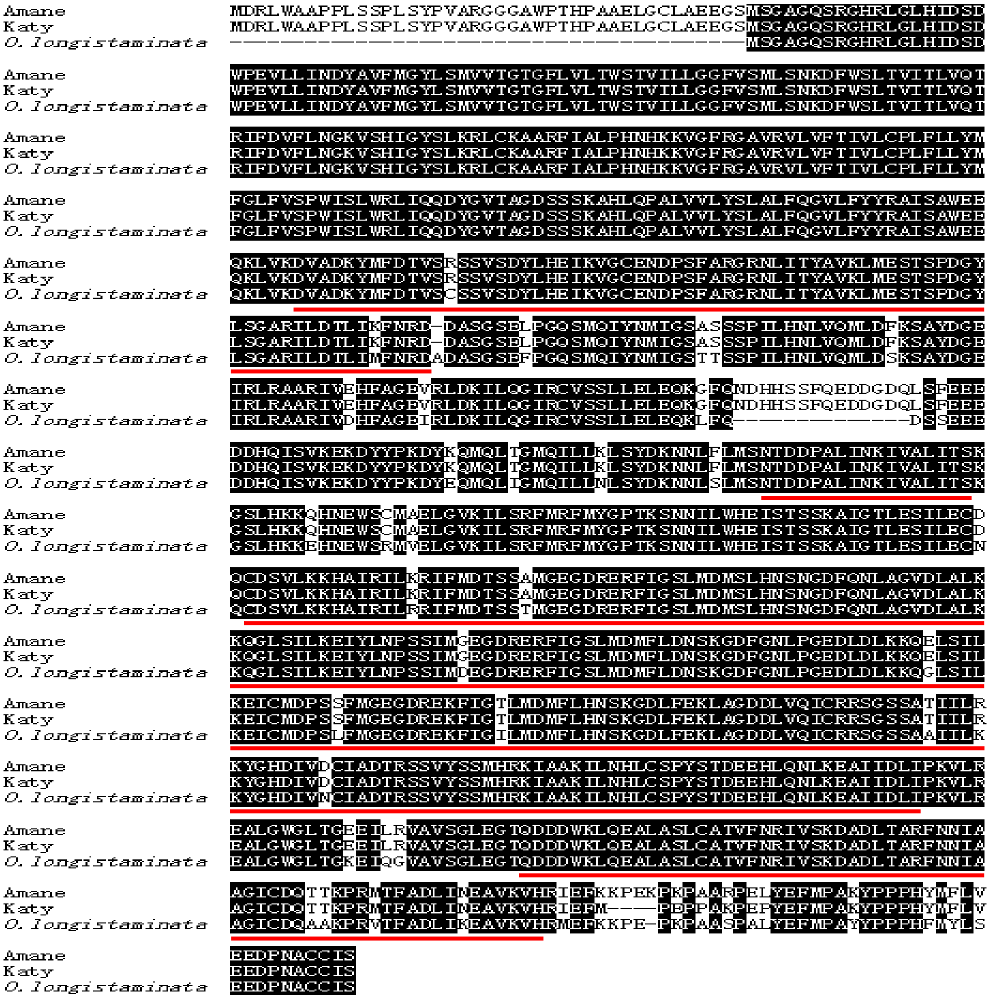

Supplementary Figure 9. Amino acid assemblies showing protein variation among selected Ptr variants including Katy, Amane, and *Pi57(t)* candidate in *Oryza longistaminata*.

Identical amino acids are shown in black boxes. The predicted Armadillo (ARM) domain is indicated by red line.

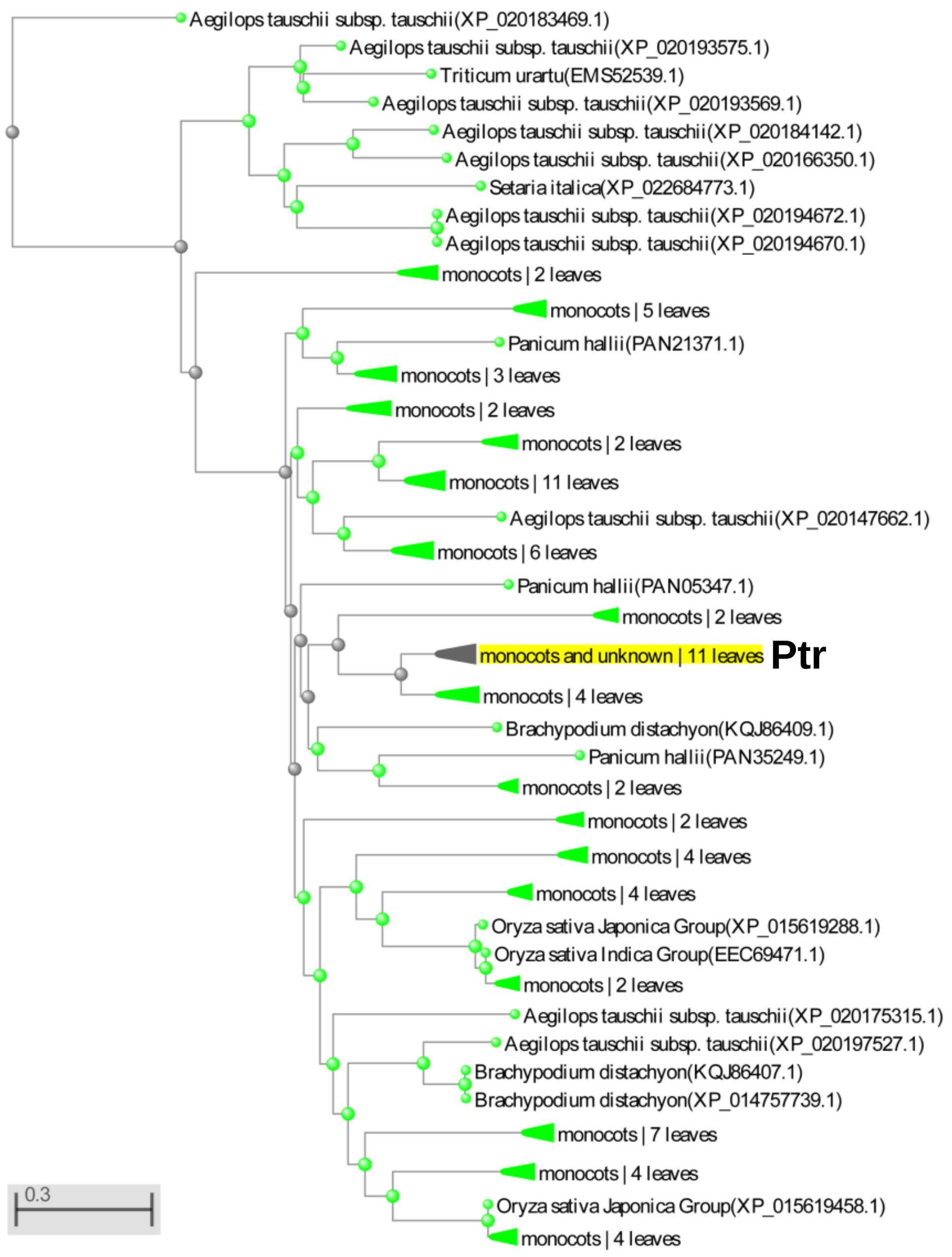

Supplementary Figure 10. Phylogenetic tree of the *Ptr* homologs in monocot plants.

The Ptr protein with 905 aa was searched in the NCBI database (https://www.ncbi.nlm.nih.gov/). Please note that one leaf means one homologous protein, in other words, 2 leaves means two homologous proteins and 11 leaves means 11 homologous proteins. The phylogenetic tree was built using Neighbor-Joining methods.

Ptr is colored in yellow and other monocots are green. The taxonomic names with protein sequence ID numbers are indicated. Microscale units are as indicated.

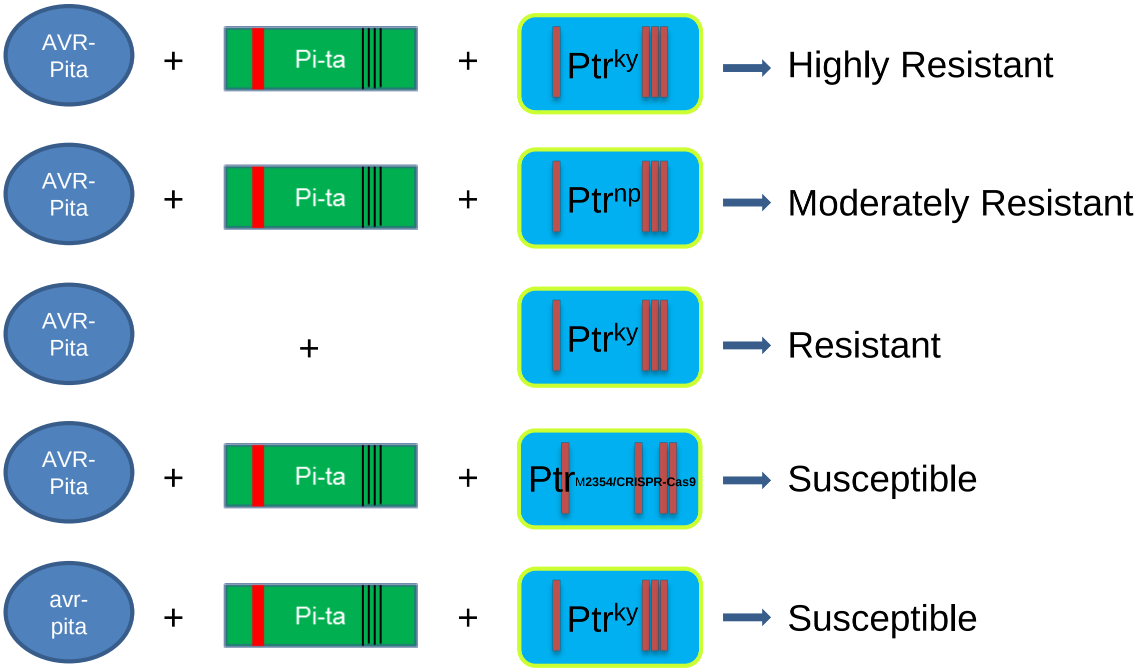

Supplementary Figure 11. The *Pi-ta* and *Ptr* genes are functionally related

Pi-ta recognizes *AVR-Pita*[31]. The resistance function of both *Pi-ta* and *Ptr* can be compromised if *M. oryzae* strain does not contain *AVR-Pita*. *Ptr* can function without *Pi-ta*, but not vice versa. Note: Red and black bars indicate nucleotide binding site-leucine-rich repeat (NBS-LRR) and pink bands indicate ARM repeats. Ptr^{ky} indicates *Ptr* gene products in Katy, Ptr^{np} ptr products in Nipponbare, $Ptr^{m2354/CRISPR\text{-}cas9}$ ptr fast neutron knockout products in M2354, and CRISPR/cas9 knockout ptr products in T2 Katy lines, respectively.

Supplementary Table 1. Sequence variations in the genomic DNA region of *Ptr*.

Position	Katy	M2354	Amane	Location/amino acid substitution type
+127	T	T	-	Intron 1
+618	C	C	T	Intron 1
+805	T	T	C	Intron 1
+1026	C	C	T	Intron 1
+1268	C	C	T	Exon 2/Synonymous
+1696	G	G	T	Intron 2
+1790	-	-	T	Intron 2
+2657	C	C	T	Intron 2
+2738	A	A	G	Intron 2
+2994	C	C	G	Intron 2
+3411	C	C	T	Intron 2
+3644	T	T	C	Intron 2
+3903	C	C	T	Intron 2
+4020	C	C	T	Intron 2
+4033	G	G	A	Intron 2
+4102	-	-	A	Intron 2
+4228	G	G	A	Intron 2
+4600	T	T	C	Exon 3/Synonymous
+5918-+5919	GG	--	GG	Exon 3/Frame shift
+6383	T	T	-	Intron 3
+6925-+6926	TG	TG	AA	Exon 4/Nonsynonymous(M/K)
+6927	------------	------------	AAACCAGAAAAA	Exon 4/In-frame (KPEK)
+6929	T	T	A	Exon 4/Synonymous
+6930	G	G	A	Exon 4/ Nonsynonymous (E/K)
+6936	C	C	G	Exon 4/ Nonsynonymous (P/A)
+6943	A	A	G	Exon 4/ Nonsynonymous (K/R)
+6952	C	C	T	Exon 4/ Nonsynonymous (P/L)

The positions of DNA polymorphism are noted based on the *Ptr* genomic DNA sequence in Katy and begin from the start codon ATG (Katy, Genbank accession number: MG385185 and Amane, MG385186. M2354 sequence is identical to that of Katy except two base deletion at 5918 resulting in frameshift whereas 23 SNPs and four InDels including a 12 bp InDel between Katy and Amane. The single letter code of amino acid is indicated followed their corresponding DNA. '- ' indicates nucleotide absence.

Supplementary Table 2. Co-segregation analysis of disease reaction and InDels of the *Ptr* gene.

**Population**	**Phenotype (Allele size)**			**χ^2**	***P***
	Homozygous resistant	Heterozygous resistant	Susceptible		
M2354/Katy $F_{2:3}$ lines	73 (211 bp)	146 (209/211 bp)	81(209bp)	0.640	0.726
Katy/Amane $F_{2:3}$ lines	105(212 bp)	208 (212/223 bp)	87 (223bp)	2.260	0.323
M2354/Amane F_2	0	0	160 (42 (209 bp), 77(209/223 bp), and 41(223 bp))		

Three- to four-leaf seedlings were inoculated with *M. oryzae* race (isolate) IB49(ML1). Disease reactions were scored seven days post-inoculation (DPI) as previously described. For the Z11 InDel marker, Katy resistance allele size is 211 bp and M2354 susceptible allele size is 209 bp. For the Z12 InDel marker, the genotype of Katy resistance allele size is 212 bp and Amane susceptible allele size is 223 bp. A ratio of 3:1 was tested. χ^2 (chi-square) and *P* (probability) was calculated by Microsoft office excel 2010. The results indicate that *Ptr* is a single dominant nuclear gene; and the 2 bp deletion in M2354 and the 12 bp insertion of the *ptr* allele in Amane compared with that of Katy co-segregated with blast susceptibility.

Supplementary Table 3. Assay of *Ptr* mutant rice line M2354 with isogenic fungal strains that identify *Pi-ta* and a second *R* gene associated with *Pi-ta2* specificity[16].

Variety	O-137 (*AVR-Pita, avr-pita2-*)	CP3337 (*avr-pita, avr-pita2-*)	*Pi-ta*	O-135 (*avr-pita, AVR-Pita2*)	CP753 (*avr-pita, avr-pita2-*)	*Pi-ta2*-specific *R* gene
Yashiro-mochi	AVR	vir	+	vir	vir	-
K1	AVR	vir	+	vir	vir	-
Tadukan	AVR	vir	+	AVR	vir	+
Tetep	AVR	vir	+	AVR	vir	+
Reiho	AVR	vir	+	AVR	vir	+
Katy	AVR	vir	+	AVR	vir	+
M2354	vir	vir	+	vir	vir	-
YT16	vir	vir	-	vir	vir	-
C101A51	vir	vir	-	AVR	AVR	-

Specifically, strain CP3337 is a spontaneous mutant of O-137 (contains *AVR-Pita*) that was selected for gain of virulence on *Pi-ta* rice variety Yashiro-mochi, and it had lost a functional *AVR-Pita* gene. Mutant strain CP753 derived from strain O-135 (lacks *AVR-Pita*) was selected for gain of virulence on *Pi-ta2* rice variety Reiho, identifying a second *AVR* gene specific for *Pi-ta2*. '+' indicates *R* and *AVR* gene present and '-' indicates absence. C101A51 contains the independent *R* gene *Pi-2*.

Supplementary Table 4. Genotype analysis of recombinant inbred lines of Saber with Cybonnent and parents.

Rice variety and RIL line	Gene-specific markers				Diagnostic microsatellite markers							
	Pi-km (RM 224)	*Pi-ta*	*Ptr*	*Pi-b* (RM 208)	RM 1339	RM 247	RM 24011	RM 228	RM 190	RM 215	RM 232	RM 154
Saber	139(R)	216(S)	223(S)	177(R)	126	163	420	114	126	154	156	199
Cybonnet	139(R)	215(R)	212(R)	163(S)	126	135	420	114	113	150	158	179
S/C272	139(R)	216(S)	212(R)	163(S)	126	163	420	114	113	150	158	199
S/C324	139(R)	216(S)	212(R)	163(S)	126	163	420	114	113	154	158	NA
S/C353	139(R)	216(S)	212(R)	163(S)	126	163	420	114	113	150	158	199

SSR marker RM224 for *Pi-km*, RM208 for *Pi-b*, and *Pi-ta* and *Ptr* gene-specific markers were used to determine the existence of *R* genes. Eight diagnostic microsatellite markers were used to verify the identity of parents. These data show that all 3 SC RILs contain *Pi-km* and *Ptr* but lack *Pi-b* and *Pi-ta*. *Pi-km* virulent race/isolates (in Fig. 3a and Table 1) should be able to determine resistance specificity and spectrum of the *Ptr* gene. *R* indicates resistant allele and S indicates susceptible allele, respectively. NA means data is not available.

Supplementary Table 5. Genotyping and phenotyping of M202/SC272 F_2 lines with diagnostic DNA markers with indicated physical locations in megabases.

Line ID	RM3246 9.09	*Pi-ta* marker 10.6	XW196 10.79	*Ptr* marker (Z12) 10.8	Phenotype IB-49 (ML1)
M202/SC272_1	193	216	203	223	5
M202/SC272_2	190/193	216	203/205	212/223	2.5
M202/SC272_3	190/193	216	203/205	212/223	0
M202/SC272_4	190/193	216	203/205	212/223	2.5
M202/SC272_5	190/193	216	203/205	212/223	1
M202/SC272_6	190	216	205	212	0
M202/SC272_7	190/193	216	203/205	212/223	1.5
M202/SC272_8	190/193	216	203/205	212/223	1.5
M202/SC272_9	193	216	203	223	3
M202/SC272_10	190/193	216	203/205	212/223	0
M202/SC272_11	190/193	216	203/205	212/223	1
M202/SC272_12	190	216	205	212	1
M202/SC272_13	190	216	205	212	0
M202/SC272_14	190	216	205	212	0
M202/SC272_15	190	216	205	212	1
M202/SC272_16	190/193	216	203/205	212/223	0
M202/SC272_19	190/193	216	203/205	212/223	1
M202/SC272_20	190	216	205	212	0
M202/SC272_21	190/193	216	203/205	212/223	2
M202/SC272_22	190/193	216	203/205	212/223	1
M202/SC272_23	193	216	203	223	4
M202/SC272_24	190/193	216	203/205	212/223	0
M202/SC272_26	190	216	205	212	1
M202/SC272_27	190/193	216	203/205	212/223	0
M202/SC272_28	190	216	203/205	212/223	0
M202/SC272_29	193	216	203/205	212/223	2.5
M202/SC272_30	193	216	203	223	3
M202/SC272_31	190/193	216	203/205	212/223	2.5
M202/SC272_32	190	216	205	212	0
M202/SC272_33	190	216	205	212	1
M202/SC272_35	190/193	216	203/205	212/223	0
M202/SC272_36	190	216	205	212	0
M202 (Parent)	193	216	203	223	5
S/C 272 (Parent)	190	216	205	212	0

All F_2 progeny without *Pi-ta* and with *Ptr* were resistant to *M. oryzae* race (isolate) IB-49 (ML1). The marker allele size 216 bp is a susceptible *pi-ta* allele and the marker allele

size 212 bp is a resistant *Ptr* allele, suggesting that *Ptr* is necessary for resistance without *Pi-ta* and this resistance was inherited in tested F_2 progeny of S/C272 crossed with M202. Category data for IB-49 (ML1) used the 0-5 rating, where 0-2 indicates resistance and 3-5 indicates susceptible.

Supplementary Table 6. Sequence analysis of the functional site of *Ptr* of the fourth exon of *Ptr* in 28 rice varieties or lines.

Variety/ line	*Pi-ta/ Pita2*	*Ptr*						Pheno- type
		+6925	+6927	+6930	+6936	+6943	+6952	
Katy	+/+	T/M	------------	G/E	C/P	A/K	C/P	R
Pi No.4	+/+	T/M	------------	G/E	C/P	A/K	C/P	R
Pi No.5	+/+	T/M	------------	G/E	C/P	A/K	C/P	R
Reiho	+/+	T/M	------------	G/E	C/P	A/K	C/P	R
Tadukan	+/+	T/M	------------	G/E	C/P	A/K	C/P	R
IRBLta2-Re	+/+	T/M	------------	G/E	C/P	A/K	C/P	R
IRBLta2-Pi	+/+	T/M	------------	G/E	C/P	A/K	C/P	R
IR64	+/+	T/M	------------	G/E	C/P	A/K	C/P	R
Cybonnet	+/+	T/M	------------	G/E	C/P	A/K	C/P	R
Adny 11	+/?	T/M	------------	G/E	C/P	A/K	C/P	R
P 3084F4-56-2-2	+/?	T/M	------------	G/E	C/P	A/K	C/P	R
Sachiminori	+/?	T/M	------------	G/E	C/P	A/K	C/P	R
IRBL9-W	-/?	A/K	AAACCAGAA GAA/ KPEE	A/K	G/A	G/S	T/L	R
Pi No.1	+/-	A/K	AAACCAGAA AAA/ KPEK	A/K	C/P	A/K	C/P	S
Pi No.2	+/-	A/K	AAACCAGAA AAA/ KPEK	A/K	C/P	A/K	C/P	S
YT14	+/-	A/K	AAACCAGAA AAA/ KPEK	A/K	C/P	A/K	C/P	S
Yoshiro-mochi	+/-	A/K	AAACCAGAA AAA/ KPEK	A/K	C/P	A/K	C/P	S
MS1996-8	+/?	A/K	AAACCAGAA AAA/ KPEK	A/K	C/P	A/K	T/L	S
MO2002-13000	+/?	A/K	AAACCAGAA AAA/ KPEK	A/K	C/P	A/K	T/L	S
AR2001-111-31	+/?	A/K	AAACCAGAA AAA/ KPEK	A/K	C/P	A/K	T/L	S
Nema	+/?	A/K	AAACCAGAA AAA/ KPEK	A/K	C/P	A/K	T/L	S
Amane	+/?	A/K	AAACCAGAA AAA/ KPEK	A/K	G/A	G/R	T/L	S
Hatadawee	+/?	A/K	AAACCAGAA AAA/ KPEK	A/K	G/A	G/R	T/L	S
YT16	-/-	A/K	AAACCAGAA AAA/ KPEK	A/K	G/A	G/R	T/L	S
LTH	-/-	A/K	AAACCAGAA GAA/ KPEE	A/K	G/A	G/S	T/L	S
M202	-/-	A/K	AAACCAGAA AAA/ KPEK	A/K	G/A	G/R	T/L	S
Saber	-/-	A/K	AAACCAGAA AAA/ KPEK	A/K	G/A	G/R	T/L	S
Nipponbare	-/-	A/K	AAACCAGAA AAA/ KPEK	A/K	G/A	G/R	T/L	S

Three- to four-leaf stage seedlings were inoculated with *M. oryzae* race (isolate) IB-49 (ML1) and scored as previously described, with 0-2 as resistant and 3-5 as susceptible.

The SNP positions were noted based on the Katy *Ptr* genomic DNA (Genbank accession number MG385185) beginning from the start codon ATG in Katy. Five nonsynonymous SNPs and one InDel in the fourth exon of *Ptr* were sequenced. Letters indicate resulted amino acid due to indicated SNP or InDel. The symbols '-', '+', and '?' indicate the gene is absent, present, and unknown, respectively. The results indicate DNA variations around the 12 bp InDel of the *Ptr* gene were associated with blast resistance.

Supplementary Table 7. The *Ptr* haplotype analysis in IRRI 3K sequenced rice germplasm.

Ptr Hap ID	Total	*Pi-ta* Resis-tant	*Pi-ta* Suscep-tible	Aus	Aromatic	Indica	Admix	Japonica Admix	Temperate Japonica	Tropical Japonica	Un-assigned
Katy (Res Ptr)-16	48	48	0	1	0	33	3	0	5	2	4
Hap 1	449	2	442	9	10	397	9	3	10	10	1
Hap 2	425	0	422	25	10	56	23	43	158	107	3
Hap 3	292	8	281	8	5	156	9	30	16	67	1
Hap 4	237	4	228	16	2	186	14	6	5	8	0
Hap 5	172	0	172	0	3	16	4	22	29	97	1
Hap 6	147	136	3	2	0	136	3	3	1	2	0
Hap 7	112	2	107	3	2	69	5	10	4	19	0
Hap 8	104	1	103	64	1	29	6	0	2	2	0
Hap 9	51	0	51	0	0	4	3	2	40	2	0
Hap 10	37	0	35	2	2	29	3	0	0	1	0
Hap 11	27	0	27	2	2	2	1	1	3	16	0
Hap 12	18	3	9	1	0	14	3	0	0	0	0
Hap 13	17	0	17	1	0	14	1	0	0	1	0
Hap 14	11	0	11	1	0	1	1	0	0	8	0
Hap 15	20	20	0	0	0	18	0	0	2	0	0
Total	2167			135	37	1160	88	120	275	342	10

A total of 16 haplotype alleles were observed across 2,167 lines. Katy *Ptr* resistance allele (Hap 16) was present in 48 primary lines of *indica* ancestry. This haplotype is defined by having the same SNP profile as Katy within the exons of the *Ptr* gene and the presence of the deletion in exon 4. Haplotype 15 had the identical SNP exon haplotype as the Katy haplotype, but did not contain the exon 4 deletion. All the lines within the Katy haplotype group also contained the resistance allele of *Pi-ta*. The *Pi-ta* resistance allele was also commonly observed in other *Ptr* haplotype groups (see additional resources for information on rice variety for each haplotype and details of SNPs).

Supplementary Table 8. Analysis of genotype and phenotype of IRRI monogenic lines with *Pi-ta* and *Ptr* and their disease reaction to blast field isolates.

Rice Line	Genotyping data		*M. oryzae* race (isolate)			
	Pi-ta marker	*Ptr* marker	IB-49 (14A29_3)	IE-1 (14L7_1)	IE-1 (15A23_2)	IB-17 (15A17-1)
IRBL9-w	216	222	0.8	1.8	0.3	0.0
IRBLzt_T	216	223	0.0	0.5	0.0	0.0
IRBLks-S	216	223	3.8	4.5	1.0	2.3
IRBLkp-K60	216	223	1.0	2.2	1.0	0.5
IRBLkh-K3	216	223	1.7	1.0	0.0	0.0
LTH	216	222	3.8	4.5	4.2	4.7
Katy	215	212	0.5	0.5	0.0	0.0

Gene-specific markers of *Pi-ta* and *Ptr* were used for genotyping. The marker size for resistant *Pi-ta* is 215 bp and susceptible *pi-ta* is 216 bp, and resistant *Ptr* is 212 bp and susceptible *ptr* is 222 bp and 223 bp. Category data 0-5 scale was used for disease reaction. 0-2 indicates resistance and 3-5 indicates susceptibility. IRBL9-w is a IRRI monogenic line containing *Pi9*, IRBLzt_T contains *Pi-zt*, IRBLks-S contains *Pi-ks*, IRBLkp-K60 contains *Pi-kp*, IRBLkh-K contains *Pi-kh*[44], and Lijiangxintuanheigu (LTH) is the recurrent parent for IRRI monogenic lines[46].

Supplementary Table 9. Rice varieties used in this study.

Name	PI number	Origin	Reference
Katy	PI 527707	United States	(*7*)
M2354	NA	United States	(*12*)
Amane	PI 373335	Sri Lanka	(*32*)
Nema	PI 430981	Iraq	(*32*)
Hatadawee	PI 373122	Sri Lanka	(*32*)
Patchaiperumal	PI 373331	Sri Lanka	(*32*)
Pi No.1	PI389193	Japan	(*25*)
Pi No.2	PI291646	Japan	(*25*)
Pi No.4	PI274214	Japan	(*25*)
Pi No.5	PI389288	Japan	(*25*)
YT14	NA	United States	(*16*)
YT16	NA	United States	(*16*)
Reiho	PI 439141	Japan	(*45*)
Taducan/Tadukan	PI 280681	Japan	(*16*)
Tetep	PI 431324	Vietnam	(*16*)
K1	IRIS 4-7611	Philippines	(*16*)
C101A51	PI 597053	Philippines	(*16*)
IRBLta2-Re	NA	Philippines	(*46*)
IRBLta2-Pi	NA	Philippines	(*46*)
IRBL9-W	NA	Philippines	(*46*)
IR64	PI 497682	Philippines	(*47*)
Cybonnet	PI 636726	United States	(*48*)
Saber	PI 633624	United States	(*49*)
Adny 11	PI 433832	Nigeria	(*32*)
P 3084F4-56-2-2	PI560285	Columbia	(*32*)
Sachiminori	PI 514666	Japan	(*32*)
Yoshiro-mochi	NA	Japan	(*25*)
MS-1996-8	NA	United States	(*50*)
MO-2002-1300	NA	United States	(*50*)
AR-2001-1111	NA	United States	(*50*)
LTH	GSOR 300331	China	(*51*)
M-202	PI 494105	Unites States	(*6*)
Nipponbare	GSOR 311795	Japan	
S/C 272	NA	United States	This study
S/C 324	NA	United States	This study
S/C 353	NA	United States	This study
X1	NA	United States	This study
X55	NA	United States	This study

PI number was retrieved from USDA National Plant Germplasm System (https://www.ars-grin.gov/npgs/) or International Rice Information System (IRIS, http://iris.irri.org/).

Supplementary Table 10. Primers used for fine mapping and sequencing.

Name	Forward/ reverse sequence 5’→3’	Purpose
RM3246	F: GCCACTCATATAAGCAAATG	Fine mapping
	R: TGGTTAATGGTCAGAACCTG	
RM1047	F: ATTACAGAACCCCACTCCCC	Fine mapping
	R: CATCATCTTAGCCCCCAGTG	
RM1337	F: GTGCAATGCTGAGGAGTATC	Fine mapping
	R: CTGAGAATCTGGAGTGCTTG	
RM27941	F: GCTGTTTCGATGCGTATTTCTGC	Fine mapping
	R: GCGTGAATCTAACCGAGAAACG	
RM27973	F: CCACACTGCCCAGGATTTAAGC	Fine mapping
	R: CTGTTCCCATCATCCAAATGACC	
RM7102	F: TTGAGAGCGTTTTTAGGATG	Fine mapping
	R: TCGGTTTACTTGGTTACTCG	
RM277	F: CGGTCAAATCACCTGAC	Fine mapping
	R: CAAGGCTTGCAAGGGAAG	
W137	F: ATCCTTTCATCTGTGTTGGCCC	Fine mapping
	R: TACGCACCTTCCTATCGCTCTT	
W121	F: CGACAGGAAAAACTTGTTAGGAA	Fine mapping
	R: AGTTGTGTGTCGCTTGCTGT	
W249	F: AAGTTGTGTGTCGCTTGCTG	Fine mapping
	R: TCTGCTTTGAAGTTGGCA	
W195	F: TGTCATTAGCAGCTACGGTGGT	Fine mapping
	R: TGTTTGACCGTGGTCTTGCT	
RM 27946	F: CCTCTATGCTCGTGAGGTGTGG	Fine mapping
	R: GGAAGTTCTAATGCACGATCACG	
Z6 (*Hin*fI)	F: GCATCGGGTGTAGGCACG	Fine mapping
	R: CCCACGGAAACCTGATTATTGT	
Z14	F: TCATGCCTACATTTCTTGTTGTTT	Fine mapping
	R: GACTTGCTGAATCTCCCTGAATA	
HJ16-1	F: CATATTTGTTGGCGGATGGC	LOC_Os12g18680
	R: GGAGATCACTAAGGTTCCGACC	genomic sequencing
HJ16-2	F: GATGGGAGGAGGATGGAGC	LOC_Os12g18690
	R: AGTTAAATCGGATGAGTGTATCAAGA	genomic sequencing
HJ16-3	F: ATGCTGATCCAGTGGTTCTCG	LOC_Os12g18690
	R: CATGTCGATCAGGGAGCCAA	genomic sequencing
HJ16-4	F: AAACGGTTCATGTCTGCGACTA	LOC_Os12g18690
	R: GAAAGAATGTGGACCCAATTTACT	genomic sequencing
HJ16-5	F: ATCGCAAGGAGCAAGGTAATC	LOC_Os12g18700
	R: ACTAAGGTAAGGGCACCGAGA	genomic sequencing
HJ16-6	F: AGAACTCCACGATAATCTGAAACAA	LOC_Os12g18700
	R: GTCCCATGATGGCCCAAG	genomic sequencing
HJ16-7	F: TGGTGCGGGTTTAAGGTCTGA	LOC_Os12g18710
	R: CCCTGCCAAATCTGGAGGC	genomic sequencing
HJ16-8	F: GGTTGTAACTGATGCGAATTGTCC	LOC_Os12g18710
	R: CGGTTGGATAGCCGGGACTA	genomic sequencing
HJ16-9	F: CACATCACGCATGAACACCA	LOC_Os12g18750
	R: GGCCTCACCAGCGACATT	genomic sequencing
HJ15-18	F: TTTGTTCCCACGACCTCTATTT	*Ptr* (LOC_Os12g1879)
	R: CTTACATTTACTTCTGCGCCTTT	promoter sequencing

HJ15-19	F: AGCGGTTAGGATAGTATGGGAATA<br>R: GAGGAGGGAAAGCAGTGGTG	*Ptr* promoter sequencing
HJ15-20	F: TCAAAGTCACGAACCTCCAGC<br>R: GCCCAAGACAAGAAAGACGAAA	*Ptr* genomic sequencing
HJ15-21	F: AAACGTACAGCCCACCCA<br>R: GCAATGCGAAACTCCAAAT	*Ptr* genomic sequencing
HJ15-22	F: GGTGTTTAGCGGAGGAAGGA<br>R: TCGGCCAAAGGCACTGAT	*Ptr* genomic sequencing
HJ15-23	F: CGAATTAGCCTCGCACATAA<br>R: ACACGGACGTACTCGAAATAAA	*Ptr* genomic sequencing
HJ15-24	F: TACTAACTTGAGACGTTCGGACAC<br>R: CGACACTAACCAATCGGTCATAC	*Ptr* genomic sequencing
HJ15-25	F: AATCGGAACCAAACATACCTGA<br>R: GGATGAAGATGAGCGCCATA	*Ptr* genomic sequencing
HJ15-26	F: TCAGAGTCTACATTGCGAGCAG<br>R: TGGTTTGACCGAAGTATTACGAG	*Ptr* genomic sequencing
HJ15-27	F: TTTTAGCGGACATAGAAGTTACCC<br>R: AGGACAGATACCGACAGTGGAA	*Ptr* genomic sequencing
HJ15-28	F: TGACAGGATGGCATGTACTGAA<br>R: ATTGCTTTGCTGCTTGTTGATA	*Ptr* genomic sequencing
HJ15-29	F: GACGACGGCGACCAACTT<br>R: TTACAGTGCTTTCGGTCATTCC	*Ptr* genomic sequencing
HJ15-30	F: TCAGATGCACGAAACCATAGC<br>R: CTCCATCCGAGATAGTTTAGGAAA	*Ptr* genomic sequencing
Z12	F: TGCAGATTTGACTGCTCGGT<br>R: GGGATCTTCCTCGCCCAAA	12 bp InDel marker in *Ptr*
Z11	F: AAAGGTTCATCGGCTCCCTG<br>R: TCGCCCTTGCTGTTGTGAA	2 bp InDel marker in *Ptr*

Supplementary Table 11. Primers used for DNA constructs and transcript analysis.

Name	Forward/ reverse sequence 5’→3’	Purpose
HJ15-31	F:GTGTAGTCCCTACTCCACTGACG<br>R:CAATTCTGTTAAACACGGTTGC	qRT-PCR for *Ptr*
qActin	F:CCACTATGTTCCCTGGCATT<br>R:GTACTCAGCCTTGGCAATCC	qRT-PCR
HJ17-7	F:GACGAAGATCCTCACCGACAT<br>R:GGCAAACCACGGCTAACAATA	qRT-PCR for *Pi-ta*
HJ16-36	F:AGCCCGGAATTCATGGATAGGCTCTGGGCGGCTC<br>R:TAAGCAGTCGACTCAAGAGATACAACACGCGTTGGGA	Subcellular Localization of the full Ptr protein
HJ17-3	F:AGCCCGGAATTCATGGATAGGCTCTGGGCGGCTC<br>R:TAAGCAGTCGACCCTGGCAGATTTCCAAAGTCGCTTA	Subcellular Localization of the Ptr truncated protein in M2354
HJ16-34	F:TAAGCAGTCGACATGGATAGGCTCTGGGCGGCTC<br>R:AGCCCGGAATTCTCAAGAGATACAACACGCGTTGGGA	E3 ligase analysis of the full Ptr protein
HJ17-2	F:TAAGCAGTCGACATGTCTGCTTGGGAAGAACAGAAGCTAGTG<br>R:AGCCCGGAATTCTCAAGAGATACAACACGCGTTGGGA	E3 ligase analysis of the Armadillo (ARM) domain in the Ptr protein

Supplementary Table 12. Plasmids used in this study.

Name	Description/Reference	Purpose
pDR01	2715 bp full-length *Ptr* cDNA coding sequence (without stop codon) of Katy at the *Eco*RI and *Sal*I sites of pYBA1132	Subcellular localization
pDR02	2592bp full-length *Ptr* cDNA coding sequence (without stop codon) of Katy at the *Eco*RI and *Sal*I sites of pYBA1132	Subcellular localization
pDR03	2727 bp full-length *Ptr* cDNA coding sequence (without stop codon) of Amane at the *Eco*RI and *Sal*I sites of pYBA1132	Subcellular localization
pDR04	2604 bp full-length *Ptr* cDNA coding sequence (without stop codon) of Amane at the *Eco*RI and *Sal*I sites of pYBA1132	Subcellular localization
pDR05	1935 bp truncated *Ptr* cDNA coding sequence (without stop codon) of M2354 at the *Eco*RI and *Sal*I sites of pYBA1132	Subcellular localization
pDR06	pKO-Ptr, CRISPR/CAS9 vector for *Ptr* gene	CRISPR/CAS9
pDR07	2718 bp full-length *Ptr* cDNA coding sequence of Katy in the *Sal*I and *Eco*RI sites of pMAL-c5X	Ubiquitination assay
pDR08	2016 bp partial *Ptr* cDNA coding sequence without transmembrane domain of Katy in the *Sal*I and *Eco*RI sites of pMAL-c5X	Ubiquitination assay
pDR09	2028 bp partial *Ptr* cDNA coding sequence without transmembrane domain of Amane in the *Sal*I and *Eco*RI sites of pMAL-c5X	Ubiquitination assay
pDR10	2014 bp partial *Ptr* cDNA coding sequence without transmembrane domain of M2354 at the *Sal*I and *Eco*RI sites of pMAL-c5X	Ubiquitination assay

Supplementary Table 13. Primer sequences used for synthesizing gRNA spacers, genotyping CRISPR-edited mutants, and quantifying *M. oryzae* rDNA.

Name	sequence 5'→3'	Purpose
Ptr-Katy-PS1	TACTCCCTGGCCCTGTTCCA (GGG)	CRISPR/CAS9 Protospacer adjacent motif (PAM) sequence
Ptr-Katy-PS2	TGGTCCTGTATGGCAGAGCT (CGG)	
Ptr-PS1-gF	cg GGTCTC C AGATGCATAGA gttttagagctagaa	Assembly of PTG
Ptr-PS1-tR	ta GGTCTC A ATCTTTCAACCTGA tgcaccagccggg	
Ptr-PS2-gF	cg GGTCTC C ATGGCAGAGCT gttttagagctagaa	Assembly of PTG
Ptr-PS2-tR	ta GGTCTC A CCATACAGGACCA tgcaccagccggg	
Ptr-F1	TCACCATCGTCTTGTGTCCG	Screening of lines
Ptr-R1	ACCCTTGTTTCTTGAGCGCC	
MG_28s-qF	TACGAGAGGAACCGCTCATTCAGATAATTA	qPCR for Fungal rDNA
MG_28s-qR	TCAGCAGATCGTAACGATAAAGCTACTC	

Supplementary References

44. Kiyosawa, S. Establishment of differential varieties for pathogenicity test of rice blast fungus. *Rice Genet. Newsl.***1**, 95-97 (1984).

45. Imbe, T. & Matsumoto, S. Inheritance of resistance of rice varieties to the blast fungus strains virulent to the variety "Reiho". *Jpn. J. Breed.* 35, 332-339 (1985).

46. Tsunematsu, H. et al. Development of Monogenic Lines of Rice for Blast Resistance. *Breed. Sci.* **50**, 229-234 (2000).

47. Bonman, J. M., Estrada, B. A. & Bandong, J. M. Leaf and Neck Blast Resistance in Tropical Lowland Rice Cultivars. *Plant Dis.* **73**, 388-390 (1989).

48. Gibbons, J. W. et al. Registration of 'Cybonnet' rice. *Crop Sci.* **46**, 2317-2318 (2006).

49. McClung, A.M. et al. Registration of 'Saber' rice. *Crop Sci.* **44**, 693-694 (2004).

50. Lee, S. et al. Molecular evolution of the rice blast resistance gene *Pi-ta* in invasive weedy rice in the USA. *PLoS ONE* **6**, e26260 (2011).

51. Ling, Z., Mew TV, Wang, J. & Lei, C. Development of near isogenic lines as international differentials of the blast pathogen. *Int. Rice Res. Notes* 20, 13-14 (1995).
