# Supplementary material for: The rice blast resistance gene Ptr encodes an atypical protein required for broad-spectrum disease resistance
Source: Nat Commun. 2018 May 23;9:2039. doi: 10.1038/s41467-018-04369-4 (PMC5966436; doi:10.1038/s41467-018-04369-4)
Supplement: Supplementary file 2 — Description of Additional Supplementary Files [file 41467_2018_4369_MOESM2_ESM.pdf]

Description of Additional Supplementary Files

Supplementary Data 1.

Disease reactions of Katy, M2354, and LTH to 389 diverse M. oryzae isolates.

Supplementary Data 2.

Additional resource of the *Pi-ta* allele with 4 diagnostic SNPs and *Ptr* haplotype in each of sequenced rice varieties in supplementary table 7 and figure 5 (RES and SUS indicate resistance and susceptible respectively).
